# Metabolic-Immune Reprogramming via CuZnS@BSA Nanoregulators to Overcome Resistance in Triple-Negative Breast Cancer

**DOI:** 10.7150/thno.134131

**Published:** 2026-07-22

**Authors:** Jingyi Yang, Qi Li, Pi Zhao, Zixin Luo, Zhaokai Wang, Rui Yang, Min Zhou, Jie Zhou, Daozhen Chen, Yu Chen

**Affiliations:** 1 Affiliated Women’s Hospital of Jiangnan University, Wuxi School of Medicine, Jiangnan University, Wuxi 214002, China; 2 Wuxi Higher Health Vocational Technology School, Wuxi, Jiangsu 214028, China; 3 Wuxi School of Medicine, Jiangnan University, Wuxi, Jiangsu 214122, China

**Keywords:** triple-negative breast cancer, cuproptosis, immunogenic cell death, stimulator of interferon genes (STING), cancer immunotherapy

## Abstract

**Rationale:**

Since the therapeutic resistance of triple-negative breast cancer (TNBC) is mainly attributable to excessive glutathione (GSH) accumulation and its ‘cold’ immune landscape, we designed biomimetic CuZnS@BSA nanoregulators that exploit a pH-triggered ‘disarm-and-attack’ cascade, thereby initiating a well-defined, sequential therapeutic process in the acidic tumor microenvironment.

**Methods:**

Biomimetic CuZnS@BSA nanoclusters were synthesized via a self-assembly method. Their pH-responsive release kinetics and synergistic therapeutic mechanisms (GSH depletion, ROS generation, and cuproptosis) were systematically evaluated *in vitro* using 4T1 cells. *In vivo* anti-tumor efficacy, immune microenvironment remodeling, and anti-metastatic effects were investigated in subcutaneous and lung metastasis TNBC mouse models, both alone and in combination with PD-L1 blockade.

**Results:**

The platform first releases H_2_S to deplete intracellular GSH, thus removing the major antioxidant defenses of the tumor, then follows with the release of Cu^2+^ to induce cuproptosis, which effectively bypasses the apoptosis resistance commonly seen in TNBC. In addition, the released Zn^2+^ acts as an immune modulator by promoting the recognition of leaked mitochondrial DNA. This activates the cGAS-STING signaling pathway, and *in vivo* experiments clearly showed that it remodels the tumor microenvironment in a highly favorable manner, characterized by increased CD8^+^ T cell infiltration and enhanced dendritic cell maturation.

**Conclusion:**

Combining this nanoregulator with PD-L1 blockade led to potent suppression of both subcutaneous tumor growth and lung metastasis, thus providing a direct, elegant link between metabolic reprogramming and systemic immune activation for TNBC therapy.

## Introduction

Triple-negative breast cancer (TNBC) constitutes about 15-20% of all clinical breast cancer cases, but it is notable for being responsible for a far greater proportion of breast cancer deaths than its prevalence would suggest, owing to its aggressive clinical phenotype and the long-standing paucity of druggable therapeutic targets 1, 2. Because TNBC tumors do not express hormone receptors or HER2, traditional chemotherapy remains the standard treatment. Although the clinical standard of care is well established 3, it is widely recognized that the efficacy of current treatments for TNBC is severely limited by the distinctive physiological features of this disease: an acidic local microenvironment, active immune evasion, and the accumulation of high levels of glutathione (GSH), which serves as a potent antioxidant barrier 4-6. Therefore, immune-based approaches are a promising avenue. Although checkpoint inhibitors have shown promise in certain patient groups, their clinical benefit is frequently limited because effector immune cells cannot penetrate the tumor parenchyma, a phenomenon known as immune exclusion, which is directly driven by severe metabolic stress and the dense GSH barrier 7. Since the present therapies for TNBC are limited to killing malignant cells, the next generation of TNBC therapies must move beyond that and instead aim to actively destroy the tumor's integrated metabolic and immunological defenses.

Recent progress in metallobiology has clearly and convincingly established cuproptosis as a highly promising new strategy for overcoming drug-resistant TNBC, in sharp contrast to classical apoptosis, which acts on the cell membrane, since cuproptosis directly targets lipoylated enzymes of the tricarboxylic acid (TCA) cycle to induce lethal proteotoxic stress 8. Thus, therapeutics based on this copper-driven process can bypass the conventional survival mechanisms of TNBC. There is a clear and important obstacle to progress, which the authors discuss very nicely in the context of clinical translation: because TNBC cells have exceptionally high intracellular GSH levels, these molecules avidly bind and chelate incoming Cu²⁺, thereby blocking the formation of toxic copper complexes needed for DLAT oligomerization and cell death 9. Hence, GSH depletion is not merely a contributing factor but an absolute requirement for cuproptosis to occur. Equally significant is the established role of Zn²⁺ as a regulator of the innate immune response, which is to amplify cGAS-STING signaling and thus directly link local tumor cell death to systemic immune activation 10.

Though the existing results are quite clear, there is no doubt that constructing a single, clinically useful platform capable of precisely controlling the spatial and temporal sequence of the events under study is extremely difficult 11, 12. Therefore, the authors very cleverly designed a biomimetic CuZnS@BSA nanoregulator that carries out a well-ordered therapeutic cascade: it releases H₂S upon entering the acidic tumor microenvironment. This molecule acts as a metabolic sensitizer that rapidly consumes GSH and therefore efficiently disrupts the tumor’s antioxidant defense system, making the initial step of clearing GSH an absolute prerequisite. Once GSH is cleared, the liberated Cu²⁺ induces severe cuproptosis, while the system simultaneously releases Zn²⁺, which functions as an immune amplifier by directly boosting the cGAS-STING signaling axis 13, a pathway that is naturally activated by mitochondrial DNA ( mtDNA ) released from dying tumor cells. By packaging all these sequential events within a biocompatible protein carrier, the authors have designed a neat, elegant strategy linking local metabolic reprogramming with systemic immune activation to treat metastatic TNBC 14.

## Results

### Synthesis and characterization of CuZnS@BSA

**Figure 1:**
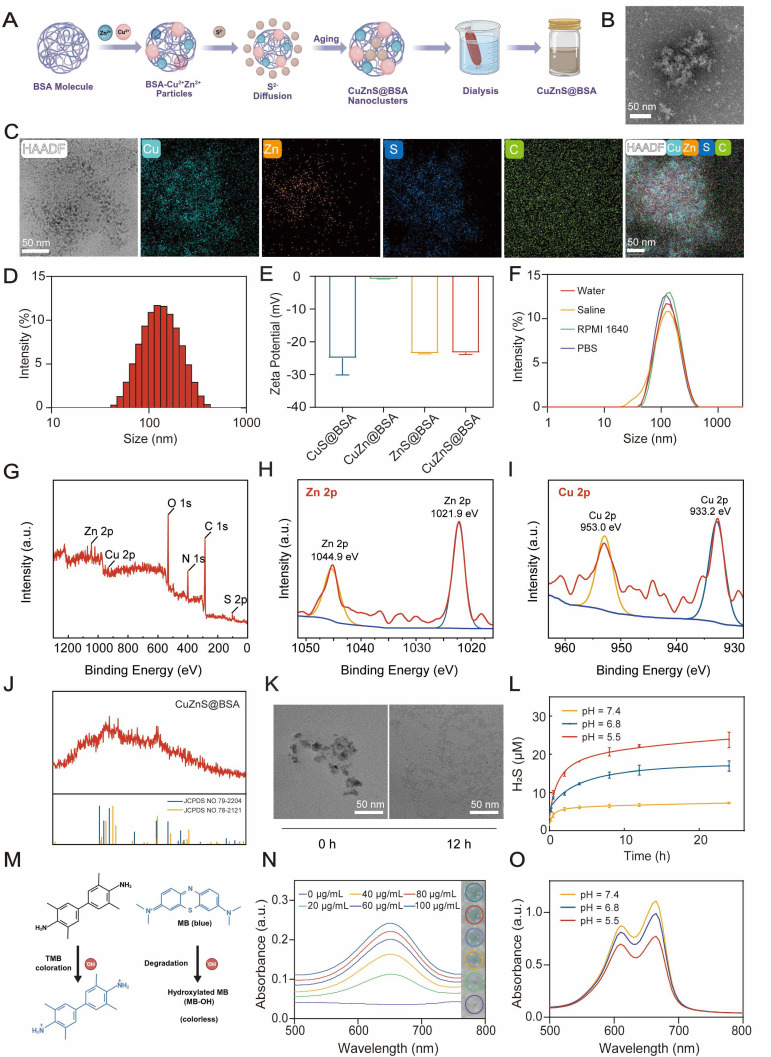
** Synthesis and characterization of CuZnS@BSA. (A)** Illustrative diagram of the synthesis pathway. **(B)** Representative negative-staining TEM image (Scale bar: 50 nm).** (C)** Morphology and EDS mapping of CuZnS@BSA (Scale bar: 50 nm). **(D)** DLS-based size distribution (n = 3). **(E)** Zeta potential comparison among BSA-stabilized variants (CuS, CuZn, ZnS, and CuZnS). (f) Hydrodynamic size stability in various media.** (G)** XPS survey spectrum. **(H, I)** High-resolution Cu 2p and Zn 2p XPS spectra. **(j)** XRD pattern of the nanoclusters.** (K)** TEM morphology of CuZnS@BSA in pH 6.0 PBS. **(l)** pH-responsive H_2_S release kinetics (n = 3, mean ± SD). **(M)** Schematic representation of the ·OH detection mechanism via TMB oxidation (blue) and MB degradation (colorless). **(N)** Time-dependent TMB oxidation by CuZnS@BSA (n = 3).** (O)** pH-dependent MB degradation efficiency (n = 3).

Because of the therapeutic difficulties inherent in TNBC, we developed biomimetic CuZnS@BSA nanoclusters by a simple and elegant self-assembly method, namely the direct coordination of sulfur (S^2-^), zinc (Zn^2+^), and copper (Cu^2+^) ions within bovine serum albumin (BSA) templates (Figure 1A). As a result, the obtained nanoplatform is ideal for multimodal treatment and exhibits remarkable therapeutic sensitivity. Because the particles were expected to respond to the acidic tumor microenvironment (TME), we first determined their particle morphology by negative-staining transmission electron microscopy (TEM) (Figure 1B), which clearly showed the contours of the BSA framework and the densely packed CuZnS metal cores embedded within the protein matrix. In a following step, elemental mapping was performed to confirm that carbon, sulfur, zinc, and copper were uniformly distributed. The particles are uniformly distributed throughout the structures (Figure 1C), and from dynamic light scattering (DLS) measurements it is clear that we have an average hydrodynamic diameter of 150 nm (Figure 1D) and a surface charge of -23 mV (Figure 1E). Since the dispersibility of the material in several important physiological fluids, namely cell culture medium, saline, phosphate buffered saline (PBS), and water, was systematically tested and found to be excellent, and because the material is stable under all tested conditions, its suitability for *in vivo* applications is clearly and convincingly established (Figure 1F). Structural characterization further confirms this.Since the CuZnS@BSA composite is fully amorphous (Figure 1J) and the BSA matrix effectively suppresses crystal growth, it naturally degrades faster in acidic media, making X - ray photoelectron spectroscopy (XPS) an ideal tool to determine its elemental composition. Therefore, initial survey scans were carried out, and the results unambiguously confirmed that all desired elements had been incorporated into the nanoplatform (Figure 1G). The high-resolution Cu 2p (Figure 1H) and Zn 2p (Figure 1I) spectra gave unambiguous, direct evidence for bimetallic coordination and the oxidation states of the metals. To test environmental responsiveness, the nanoclusters were incubated in pH 6.0 PBS solution, and subsequent TEM imaging showed rapid, acid-induced disintegration (Figure 1K), hence conclusively demonstrating their sensitivity to the acidic TNBC microenvironment.

The generation profiles of H_2_S were analyzed to study the release kinetics of the active components (Figure 1L), and it was clearly established that under neutral physiological conditions (pH 7.4) the leakage of H_2_S was negligible. Acidic conditions, on the other hand, caused a rapid, marked release, reaching 12.9 × 10^-6^ M at pH 6.8 after 24 h and rising to 23.7 × 10^-6^ M at pH 5.5. We applied the Higuchi model (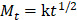) to analyze the underlying kinetics and thus determined a release rate constant (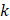) that showed an unambiguous, well-defined dependence on acidity: it went from 0.03656 at pH 7.4 to 0.10629 at pH 6.8 and reached a maximum of 0.16416 in the pH 5.5 buffer. The 4.5-fold increase in the rate constant therefore clearly demonstrates the strong acid-responsive character of the nanoplatform.

We used inductively coupled plasma optical emission spectroscopy (ICP-OES) to precisely track the shedding patterns of the incorporated metals and thus unambiguously established a dual spatiotemporal responsive release mechanism (Figures S1 and S2). Specifically, Zn^²+^ escaped very rapidly from acidic buffers (pH 6.5 and 5.5) and co-temporally with H_2_S production, whereas copper leakage remained remarkably low (below 10%) under the same conditions. Therefore, the core Cu-S structure was shown to be thermodynamically stable in non-reducing acidic media. Importantly, preserving the copper center until it reaches the cytoplasm is an elegant architectural design that ensures a sufficient metal payload to effectively trigger GSH-dependent cuproptosis.

Because the remaining solid Cu-S surface retained exposed active copper sites, it reacted extremely efficiently with hydrogen peroxide via a heterogeneous Fenton-like process to generate hydroxyl radicals (OH) 16. To directly and unambiguously confirm the production of such radicals, two well-established colorimetric assays were performed: the oxidation of TMB was monitored by the increase in absorbance at 650 nm (Figures 1M, N), and the bleaching of methylene blue was tracked by the corresponding absorbance drop at 664 nm (Figures 1M, O). Hence, the data leave no doubt: pH shifts strictly regulate the CuZnS@BSA nanoclusters, and their therapeutic activity is therefore highly specific to the acidic TME.

### Synergistic ROS and apoptosis induction through coordinated redox modulation

**Figure 2:**
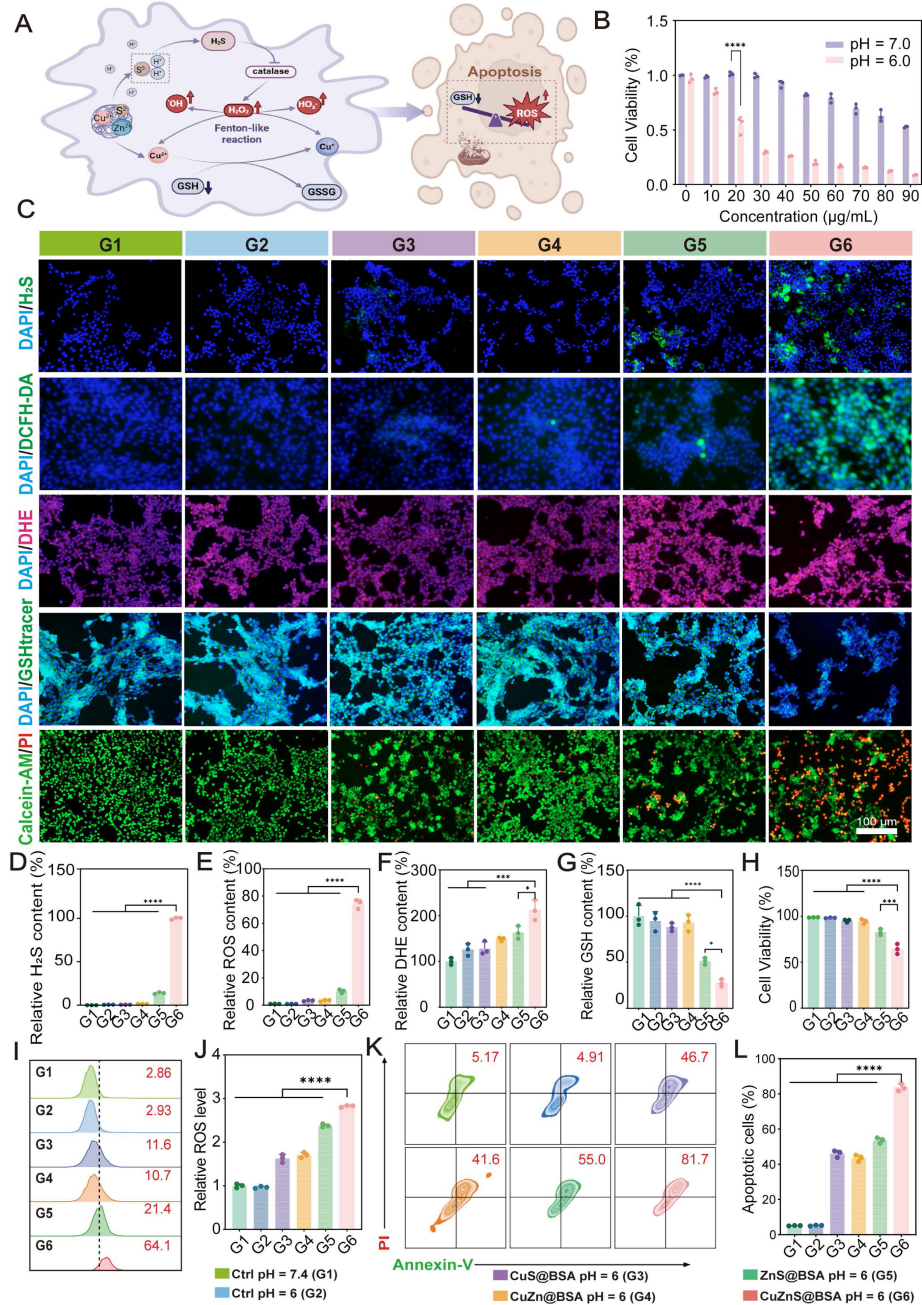
** Antitumor therapeutic efficacy of CuZnS@BSA in 4T1 cells. (A)** Illustrative mechanism of tumor microenvironment-responsive H_2_S release and therapeutic action.** (B)** Relative 4T1 cell viability across treatment groups. **(C)** Fluorescence imaging of H_2_S, ROS, ·O^2-^, and GSH, along with Calcein-AM/PI live/dead staining.** (D–G)** Semi-quantitative analysis of intracellular H_2_S, total ROS, ·O^2-^, and GSH levels.** (H)** Apoptosis quantification via flow cytometry. **(I, J)** Intracellular ROS levels and corresponding statistical analysis. **(K, L)** Flow cytometry-based apoptosis analysis and semi-quantitative statistics. Data are shown as mean ± SD (n = 3). Statistical comparisons were carried out using one-way ANOVA and Tukey’s test (^*^P < 0.05, ^**^P < 0.01, ^***^P < 0.001, ^****^P < 0.0001; n.s. = not significant).

Because the pH responsiveness of CuZnS@BSA had already been well established, it was a natural and logical next step to examine its ability to kill TNBC cells by taking advantage of the characteristic redox imbalance of this aggressive cancer type. Cytotoxicity assays with murine 4T1 cells confirmed a clear dose-dependent effect, with maximal cytotoxicity observed under acidic conditions (pH 6.0). Compared to neutral conditions (pH 7.4) (Figure 2B), the use of the inherent acidity of tumors makes these nanoregulators exceptionally attractive for targeted TNBC therapy, so we naturally proceeded to investigate the mechanistic basis of their lethality. First, the WSP-5 probe was used to unambiguously confirm that H_2_S was released only under acidic conditions (Figure 2C). This finding is particularly relevant in the context of TNBC. Since the generated H_2_S has the dual effect of rapidly depleting the tumor cell GSH pool thereby impairing its antioxidant defenses and at the same time stimulating the production of reactive oxygen species (ROS) 17, 18, it was very natural to measure intracellular ROS accumulation by DCFH-DA staining, which indeed showed a marked increase in fluorescence in the treated cells (Figure 2C). Since metal ions initiate Fenton-like reactions which then coordinate with the released H_2_S to induce massive oxidative stress, it is very natural to use DHE staining to visualize superoxide anions (·O^2-^), thereby directly and convincingly demonstrating that the platform generates a spectrum of reactive oxygen species: ·OH, ·O^2-^, and hydrogen peroxide derivatives. The article gives a clear and logical account of the internal protective mechanisms of TNBC cells (Figure 2A), supported by fluorescence imaging of GSH which unambiguously showed a sharp drop in reduced GSH levels, thereby stripping the cancer cells of essential antioxidant resources. As a result, acute cuproptotic stress activates a copper-mediated cell death pathway that circumvents the usual apoptotic checkpoints in resistant TNBC cells. Live/dead staining confirmed clearly that the CuZnS@BSA groups contained predominantly dead cells (red fluorescence) in contrast to the healthy cells (green fluorescence) in the control populations. Flow cytometry analysis then showed that 81.7% of the treated cells stained positively for Annexin V, hence demonstrating a very high overall rate of cell death. Importantly, cells display Annexin V positivity in a wide range of regulated cell death pathways, including both apoptosis and cuproptosis.

### Induction of mitochondria-targeted cuproptosis through copper-mediated metabolic disruption

**Figure 3:**
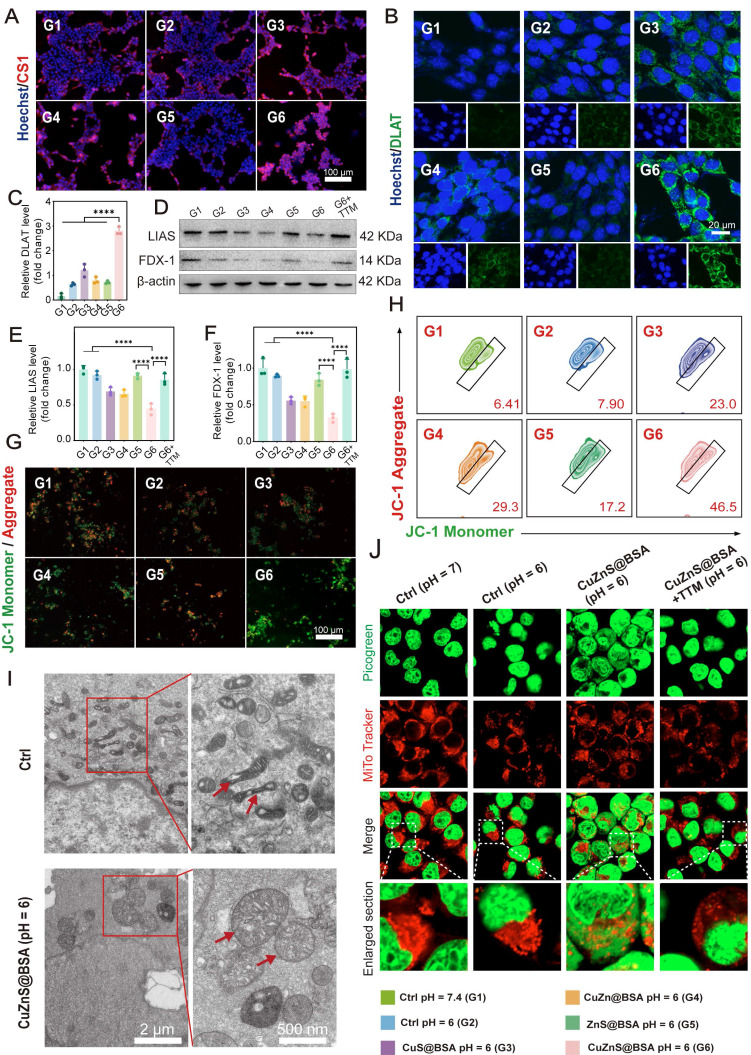
** Mechanism of copper-induced cell death in 4T1 cells. (A)** CS1 fluorescence staining of 4T1 cells post-treatment. **(B, C)** Confocal laser scanning microscopy (CLSM) images and corresponding quantification of DLAT oligomerization. **(D–F)** Western blot (WB) analysis and semi-quantitative profiling of LIAS and FDX-1 expression. **(G)** JC-1 staining showing mitochondrial depolarization. **(H)** Mitochondrial damage assessment via flow cytometry. **(I)** Bio-TEM images revealing mitochondrial structural damage (indicated by red arrows). **(J)** CLSM visualization of MitoTracker/PicoGreen co-localization to monitor mtDNA leakage (including TTM rescue group). Data are shown as mean ± SD (n = 3). Statistical comparisons were carried out using one-way ANOVA and Tukey’s test (^*^P < 0.05, ^**^P < 0.01, ^***^P < 0.001, ^****^P < 0.0001; n.s. = not significant).

We investigated whether the CuZnS@BSA platform could induce cuproptosis in conjunction with conventional apoptosis to target the mitochondrial metabolism of TNBC cells, and in order to rigorously establish a direct causal link between the observed cytotoxicity and the putative cuproptotic pathway, we carried out a series of well-designed functional rescue assays. The results clearly showed that exogenous GSH protected the cells. From the analysis of nanoregulator induced death (Figure S3), it is clearly established that copper availability determines the cytotoxic outcome, but since exogenous GSH can chelate extracellular copper ions, the present observation should be considered complementary rather than definitive evidence for intracellular GSH depletion. Therefore, we appropriately used tetrathiomolybdate (TTM) as a potent intracellular copper chelator. We have clearly and convincingly demonstrated that internal copper overload, rather than generalized oxidative stress, is the primary driver of cell death, so we used the CS1 fluorescent probe to directly track the intracellular distribution of the delivered metal. The resulting images showed robust copper accumulation in the treated cells (Figures 3A and S4), thereby unambiguously verifying that the nanoparticles entered the cells and released their cargo. After this entry, the increase in metal concentration rapidly activates the core cuproptotic pathways, and confocal microscopy clearly demonstrated DLAT oligomerization in the treated samples (Figure 3B). It is well established in the literature that such protein aggregation is the most reliable and specific biological marker for copper-induced cell death 19. Therefore, we examined the expression levels of major cuproptosis-associated proteins. We used Western blot analysis to examine LIAS and the iron sulfur cluster protein FDX1 in murine 4T1 cells treated with the nanoplatform, and clearly and convincingly showed in Figures 3D–3F that tumor cells treated with the nanoplatform had dramatically reduced levels of both LIAS and FDX1 compared to the control groups, which directly supports their mechanistic hypothesis. More importantly, addition of the specific chelator TTM restored the baseline expression of LIAS and FDX1, thereby unambiguously linking the metabolic perturbations to copper-induced toxicity 20.

Since mitochondrial membrane potential (MMP, ΔΨm) is a well-established and reliable biomarker for mitochondrial function, and its loss is closely linked to altered mitochondrial membrane permeability 21, we logically chose JC-1 fluorescent staining combined with flow cytometry to evaluate the MMP of 4T1 cells after various treatments. The results clearly showed that the CuZnS@BSA treatment group had a dramatic reduction in red fluorescence. The presence of a marked increase in green fluorescence indicates disruption of the mitochondrial membrane potential (Figure 3G-H). Since cuproptosis is associated with the accumulation of large amounts of ROS, which in turn cause mitochondrial damage and release of mitochondrial DNA (mtDNA), it was therefore logical and appropriate to analyze the mitochondrial structure of 4T1 cells treated with CuZnS@BSA using biochemical methods. From the transmission electron microscope (Bio-TEM) images it was clearly and unambiguously shown that mitochondria from 4T1 cells treated with CuZnS@BSA had marked morphological abnormalities compared to the control group: swelling, cristae shortening, and the presence of vacuoles (Figure 3I) 22. Since severe mitochondrial damage almost always leads to the release of mtDNA into the cytosol, it follows that this release is a major driver for the cGAS-STING innate immune pathway. This pathway was studied here by a well-suited dual-fluorescence co-localization approach (MitoTracker for mitochondria in red, PicoGreen for dsDNA in green), thus allowing direct, clear visualization of mtDNA leakage (Figure 3J). Untreated control samples were analyzed and showed that double-stranded DNA signals were strictly nuclear or associated with intact mitochondrial networks. Cells exposed to the CuZnS@BSA platform in a pH 6.0 medium showed dramatic mitochondrial fragmentation, with double stranded DNA distributed as clear green puncta throughout the cytoplasm, completely separated from the red fluorescent mitochondria. Importantly, addition of the specific copper chelator TTM restored mitochondrial integrity and completely blocked DNA leakage 23. The imaging data clearly and unambiguously show that massive mtDNA release is a direct consequence of copper-induced mitochondrial collapse, and more importantly, they establish that this leakage is the primary biological link between localized cuproptosis and immune activation, a point that will be discussed at length in the next section.

### Transcriptomic profiling unveils CuZnS@BSA-induced integrated stress and immune signaling pathways

**Figure 4:**
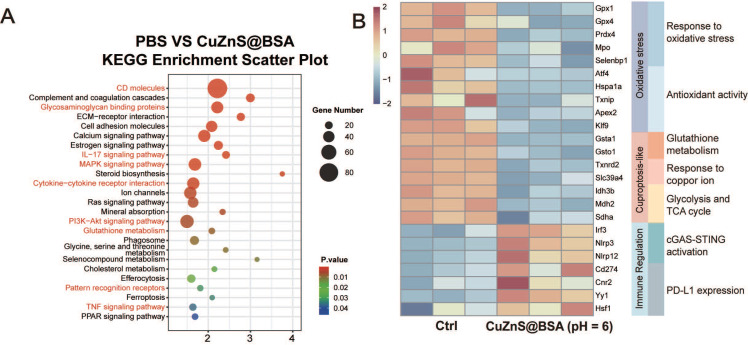
** Transcriptomic profiling unveils CuZnS@BSA-induced integrated stress and immune signaling pathways. (A)** KEGG pathway enrichment analysis. **(B)** Heatmap showing the differential expression of genes of interest (left), and the pathway associated with cuproptosis (right).

In order to systematically examine the transcriptomic changes caused by the CuZnS@BSA platform, RNA sequencing analysis was carried out, which enabled the identification of key signaling networks and the direct linkage between observed cell death phenotypes and their molecular determinants. High-throughput sequencing revealed a large number of differentially expressed genes (DEGs), and subsequent KEGG enrichment analysis clearly connected the treatment effects to tumor-associated apoptosis and immune modulation. Most importantly, the MAPK, TNF, and cytokine receptor interaction pathways were unambiguously identified in this dataset (Figure 4A).

From the expression heatmaps it is clearly and elegantly shown that prosurvival targets involved in protein protection, DNA repair, and stress adaptation are broadly suppressed, whereas immune related markers NLRP, CD274, and IRF3 are markedly upregulated (Figure 4B). Hence, the data provide direct evidence for a major reprogramming of the tumor immune microenvironment. The sequencing results further corroborate that the nanoplatform induces a synchronized transcriptional response is characterized by increased oxidative stress, major metabolic disruption, and the initiation of immune signaling, and therefore the present study elegantly connects initial metal stress and oxidant-induced cytotoxicity to the downstream events of cuproptosis and systemic immune activation. As a result, the molecular mechanisms by which localized copper and zinc interference leads to broad antitumor immunity are laid out very clearly.

### *In vitro* evaluation of cGAS-STING pathway stimulation and ICD-related responses

**Figure 5:**
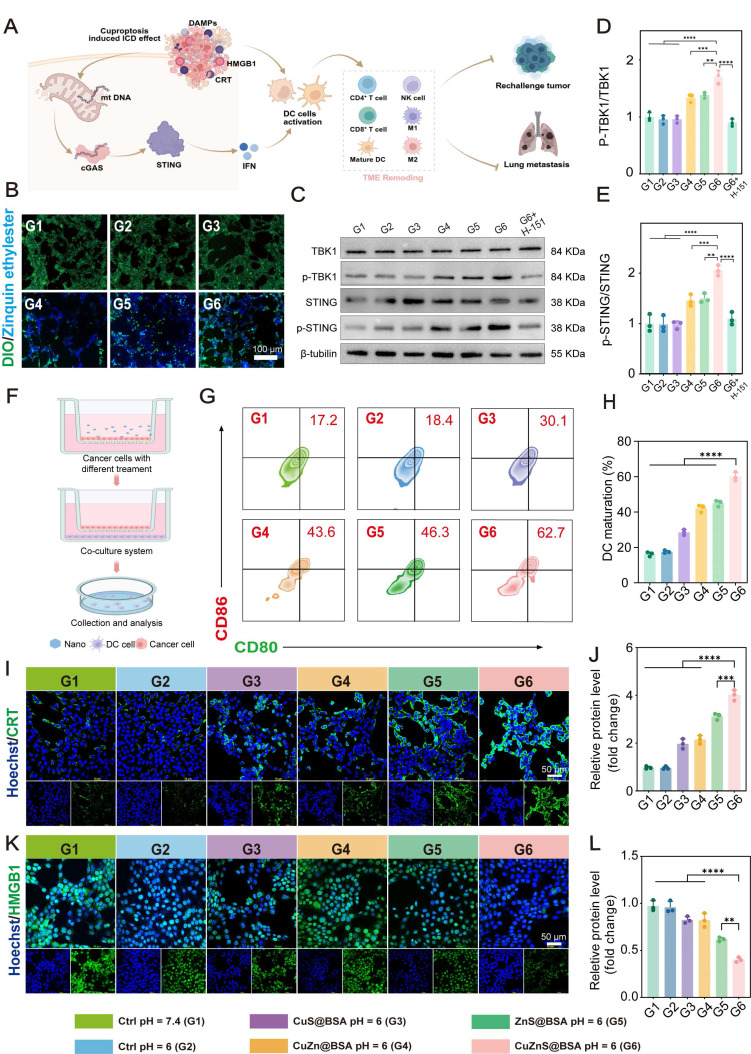
***In vitro* antitumor and cGAS/STING signaling-related experiments were performed. (A)** Schematic representation of the cuproptosis–cGAS/STING signaling pathway. **(B)** Intracellular zinc accumulation in 4T1 cells.** (C–E)** Immunoblot analysis and corresponding densitometry of STING and TBK1 phosphorylation. **(F)** Overview of the Transwell co-culture system used for 4T1 cells and dendritic cells (DCs). **(G, H)** Flow cytometry-based quantitative analysis of DC maturation (CD80^+^CD86^+^). **(I, J)** CLSM imaging and semi-quantitative analysis of CRT membrane translocation. **(K, L)** CLSM imaging and semi-quantitative analysis of HMGB1 release. Data are shown as mean ± SD (n = 3). Statistical comparisons were carried out using one-way ANOVA and Tukey’s test (^*^P < 0.05, ^**^P < 0.01, ^***^P < 0.001, ^****^P < 0.0001; n.s. = not significant).

From previous studies it is well established that CuZnS@BSA induces cuproptosis in tumor cells by perturbing mitochondrial function, thereby causing DNA damage and release. More importantly, transcriptomic analyses have uncovered a clear connection between the cGAS-STING signaling pathway and CuZnS@BSA treatment, and the released Zn²⁺ acts as an immune amplifier, potentially stabilizing the cGAS-DNA complex and thereby enhancing the sensitivity of the STING pathway to leaked mtDNA 10, which directly promotes its activation. The cGAS-STING pathway is discussed in Figure 5A, and we used a zinc-specific fluorescent probe to directly and quantitatively measure intracellular zinc accumulation after treatment, thereby showing a clear, substantial increase in zinc levels in the CuZnS@BSA-treated group (Figure 5B). Therefore, the conclusion is straightforward and well supported: the nanomaterials were taken up efficiently by cells and released zinc ions. The phosphorylation levels of TBK1 and STING were measured and thus clearly demonstrated that CuZnS@BSA activates the cGAS-STING signaling pathway (Figures 5C-E) 24. More importantly, to functionally confirm the specific involvement of this axis, the cells were co-incubated with H-151, a highly specific STING inhibitor. As expected, addition of H-151 completely blocked the nanoregulator-induced phosphorylation of both STING and TBK1. We first provided direct causal evidence for the activation of the innate immune DNA sentinel, then isolated bone marrow-derived dendritic cells (BMDCs) from mice and co-cultured them with pre-treated tumor cells in a transwell system for 48 h (Figure 5F). Since CD80 and CD86 co-stimulatory molecules are well established as reliable markers for dendritic cell maturation and activation, flow cytometry analysis clearly showed that 59.5% of the dendritic cells were. The CuZnS@BSA-treated group had a mature population in a much higher proportion than any of the other control groups (Figure 5G), therefore it is reasonable and well supported to conclude that CuZnS@BSA treatment activates innate antitumor immunity. Moreover, copper-induced mitochondrial or endoplasmic reticulum stress-mediated cell death likely overlaps with immunogenic cell death (ICD) 25. Since the process in question is critical for initiating adaptive immunity and converting ‘cold’ tumors resistant to immunotherapy into ‘hot’ tumors responsive to treatment 26, it is logical and important to determine whether CuZnS@BSA nanoparticles induce ICD. Hence, we analyzed the expression of damage-associated molecular patterns (DAMPs), namely calreticulin (CRT) and high-mobility group box 1 (HMGB1). Using immunofluorescence staining together with confocal laser microscopy, we examined 4T1 cells after co-incubation with CuZnS@BSA and clearly showed that CRT was abundantly translocated to the cell membrane (Figure 5I-J) 27, and nuclear protein HMGB1 was massively released (Figure 5K-L) 28, both of which are well-established markers of ICD in tumor cells. Therefore, a direct and compelling conclusion was drawn: CuZnS@BSA converts cell death into immune-activating signals, thus activating adaptive immunity. More importantly, the data support that CuZnS@BSA does more than induce apoptosis and cuproptosis in tumor cells *in situ*; it also bridges innate and adaptive immunity to elicit a robust, systemic antitumor response.

### Biocompatibility and biodistribution study of CuZnS@BSA

Safety is the fundamental prerequisite for the clinical application of any new material, hence before proceeding to efficacy trials, the biological distribution and safety profile of the CuZnS@BSA platform were rigorously evaluated. Hemolysis assays were first used to assess blood compatibility, revealing an exceptionally low hemolysis rate below 5% (Figure S5), which therefore unequivocally establishes that the nanoregulators are compatible with circulating red blood cells. Since safety needed to be rigorously established, appropriate toxicity studies were carried out in healthy mice, and it was clearly shown that the material produced no detectable adverse effects: animal body weight, routine serum biochemical parameters, and organ histology were all within normal ranges (Figures S6–S8). Therefore, the biocompatibility of the nanoplatform is convincingly demonstrated, and its use in systemic applications is fully justified.

**Figure 6:**
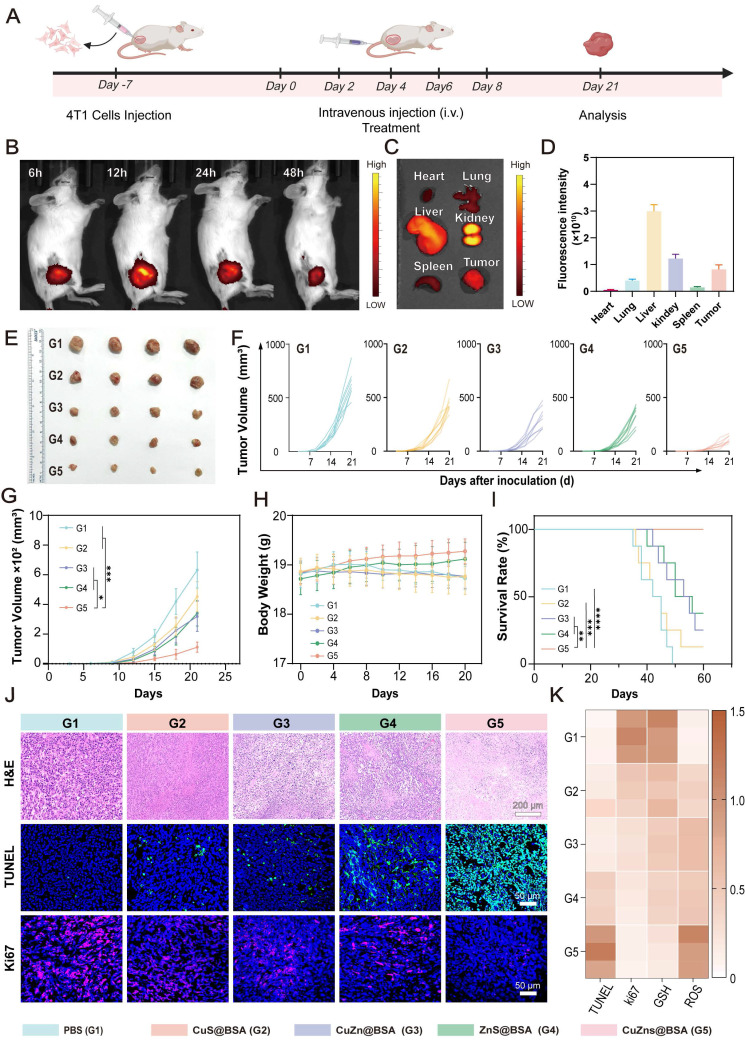
***In vivo* therapeutic efficacy and biodistribution of CuZnS@BSA. (A)** Schematic of the *in vivo* treatment protocol.** (B)** Longitudinal NIR-II fluorescence imaging of tumor-bearing mice post-injection.** (C)** *Ex vivo* fluorescence imaging of major organs and tumors harvested at 48 h. **(D)** Quantitative mean fluorescence intensity (MFI).** (E)** Representative images of excised tumor masses (n = 4). **(F, G)** Individual and average tumor growth kinetics (n = 5). **(H)** Body weight monitoring during the therapeutic period. **(I)** Kaplan–Meier survival analysis (n = 8). **(J)** Histological and immunohistochemical validation of tumor tissues (H&E, TUNEL, and Ki67; n = 3). **(K)** Semi-quantitative analysis of TUNEL, Ki67, GSH, and ROS staining intensity. Data are shown as mean ± SD (n = 3). Statistical comparisons were carried out using one-way ANOVA and Tukey’s test (^*^P < 0.05, ^**^P < 0.01, ^***^P < 0.001, ^****^P < 0.0001; n.s. = not significant).

Since the experiments were carried out on the subcutaneous 4T1 tumor model (Figure 6A), the real time biodistribution of Cy7 labeled CuZnS@BSA could be thoroughly and reliably assessed by IVIS imaging, revealing that the particles began accumulating at the tumor site 1 h after injection, the fluorescent signal increased continuously, reached a peak at 12 h, and remained very high at 48 h (Figure 6B). The following organ dissections were then performed to confirm this. The live observations were nicely corroborated: tumor uptake reached a high level of 0.82 × 10^10^ ps^-1^cm^-2^sr^-1^, which was significantly greater than that observed in the heart, lungs, or spleen (Figures 6C and 6D). Thus, the tumor targeting efficiency of the platform was clearly demonstrated and is most plausibly explained by the enhanced permeability and retention (EPR) effect. As expected, the liver showed the highest overall signal intensity, consistent with the well-characterized metabolic clearance pathways for foreign nanomaterials.

### Efficacy study of CuZnS@BSA *in vivo*

Since the safety and tumor-targeting properties of CuZnS@BSA had been established, the next logical step was to evaluate its therapeutic efficacy in a mouse model of TNBC, and the results were very clear and impressive: monotherapy with the nanoregulator reduced the average tumor mass to only 20% of the values seen in the PBS control group (Figure S9). Thus, the platform effectively suppressed primary tumor growth. The intervention described in Figures 6E to 6G was carried out without causing any detectable treatment-related body weight loss in the animals (Figure 6H), and survival data therefore provided clear, unambiguous support for its potent antitumor effect: the Kaplan Meier curves showed a statistically significant prolongation of overall lifespan in treated mice (Figure 6I). Since this was expected, histological examination of the excised tumors showed quite clearly marked nuclear condensation following treatment. The morphological change described is fully consistent with extensive cellular destruction occurring via several overlapping pathways, and immunofluorescence staining provided clear, unambiguous evidence: the tissues showed severe suppression of Ki67 expression (red fluorescence) together with a dramatic upregulation of DNA damage markers (green fluorescence) (Figure 6J). More importantly, CuZnS@BSA treatment was found to reduce GSH levels in tumor tissues while increasing ROS levels relative to other groups (Figures S10, Figure 6K). Therefore, it is convincingly shown that CuZnS@BSA has both direct cytotoxic effects on tumor cells and the capacity to modulate the tumor microenvironment.

### Tissue profiling provided initial insights into the underlying immune mechanism

**Figure 7:**
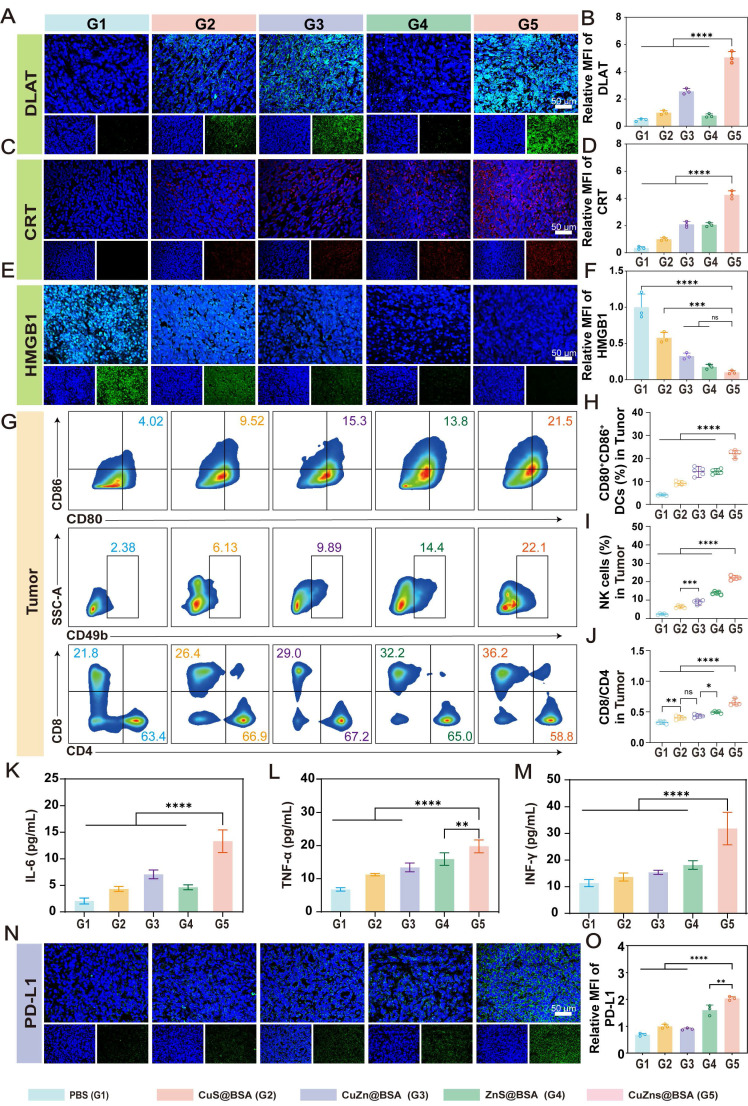
** The CuZnS@BSA complex synergistically enhanced immune activation through the integration of cuproptosis and the cGAS-STING pathway. (A, B)** Immunofluorescence analysis of DLAT expression in tumor tissues (n = 3).** (C, D)** CRT membrane translocation and relative fluorescence intensity quantification (n = 3). **(E, F)** CLSM visualization and semi-quantitative analysis of cytosolic HMGB1 release (n = 3). **(G)** Representative flow cytometry profiles of mature dendritic cells (DCs), NK cells, and the CD8⁺/CD4⁺ T cell ratio. **(H–J)** Quantitative assessment of DC maturation frequency, NK cell infiltration, and CD8⁺/CD4⁺ T cell ratio in tumor tissues (n = 5). **(K–M)** Serum levels of IFN-γ, TNF-α, and IL-6 determined by ELISA (n = 5). **(N, O)** Immunofluorescence staining and corresponding relative MFI analysis of PD-L1 expression in tumor sections (n = 3). Data are shown as mean ± SD (n = 3). Statistical comparisons were carried out using one-way ANOVA and Tukey’s test (^*^P < 0.05, ^**^P < 0.01, ^***^P < 0.001, ^****^P < 0.0001; n.s. = not significant).

**Figure 8:**
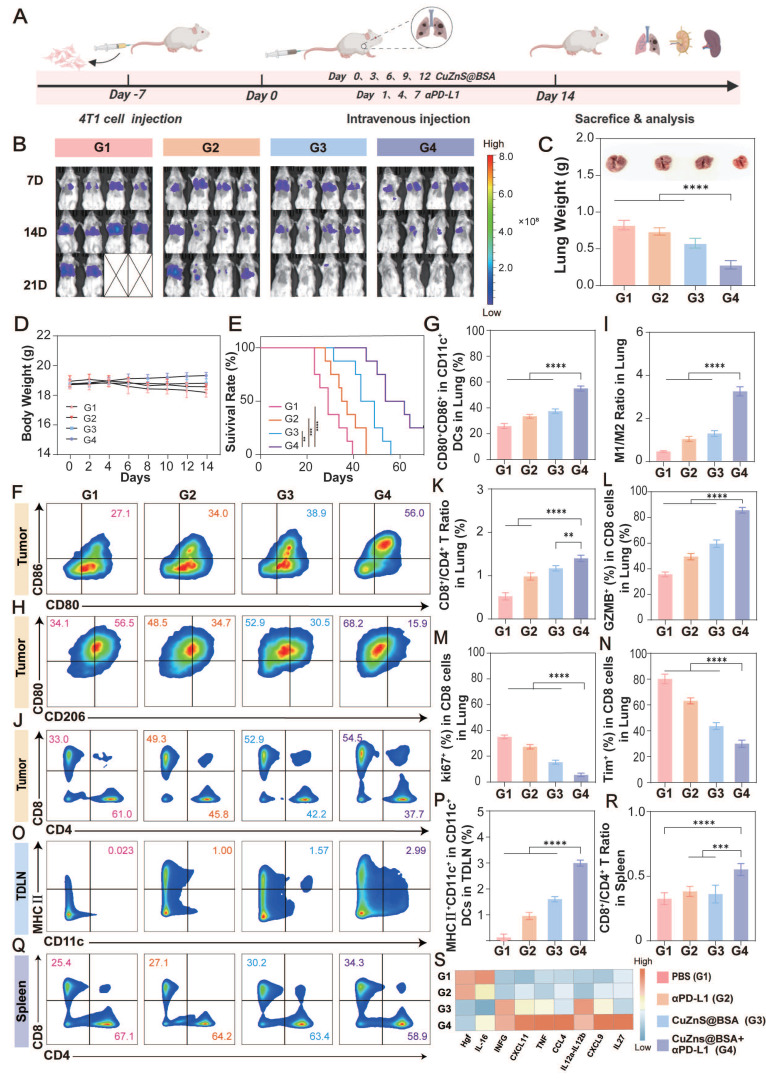
** CuZnS@BSA combined with αPD-L1 synergistically inhibited breast cancer lung metastasis. (A)** Schematic of the anti-metastatic therapeutic protocol. **(B)** Bioluminescence imaging of lung metastasis progression (days 7, 14, and 21; n = 4). **(C)** Lung metastatic nodule weight at day 21 (n = 4). **(D)** Body weight monitoring (n = 6). **(E)** Kaplan–Meier survival analysis (n = 8).** (F, G)** Flow cytometric quantification of DC maturation in lung tissues. **(H, I)** M1/M2 macrophage polarization ratio in pulmonary tissues. **(J, K)** CD8⁺/CD4⁺ T cell ratio in the lungs.** (L–N)** Frequency of GzmB⁺, Ki67⁺, and Tim3⁺ subsets within the lung-infiltrating CD8⁺ T cell population.** (O, P)** DC maturation profile in tumor-draining lymph nodes (TDLNs). **(Q, R)** CD8⁺/CD4⁺ T cell ratio in the spleen. **(S)** Serum cytokine profiling via Olink multiplex assays. Data are shown as mean ± SD (n = 3). Statistical comparisons were carried out using one-way ANOVA and Tukey’s test (^*^P < 0.05, ^**^P < 0.01, ^***^P < 0.001, ^****^P < 0.0001; n.s. = not significant).

Immunofluorescence experiments clearly and convincingly demonstrated cuproptosis activation due to DLAT aggregation (Figure 7A) and ICD induction via HMGB1 release and CRT exposure (Figure 7C-F). Flow cytometric analysis of tumor-infiltrating immune cells then showed promoted maturation of dendritic cells and NK cells 29, accompanied by a marked increase in the proportion of CD3⁺ CD8⁺ and CD3⁺ CD4⁺ T cells (Figure 7G). Since the CD4⁺ T cell compartment includes both helper and regulatory T cells, since we had already identified stimulating subsets and immunosuppressive subpopulations, we logically proceeded to evaluate the infiltration of regulatory T cells (Tregs) in order to systematically clarify the tumor immune microenvironment (TIME) remodeling. Immunofluorescence staining of tumor sections showed a substantial decrease in the ratio of immunosuppressive FOXP3⁺ Tregs to total CD4⁺ T cells in the CuZnS@BSA-treated group relative to PBS and single/dual-component controls (Figure S11). The targeted depletion of Tregs together with the vigorous expansion of cytotoxic CD8⁺ T cells unequivocally demonstrates reversal of the immunosuppressive TIME into an immune-active state. This conclusion is powerfully supported by ELISA data showing elevated inflammatory cytokines (IL-6, TNF-α, IFN-γ) in treated mice (Figure 8K-M) 30-32, and hence systemic immune activation 33. More importantly, PD-L1 was upregulated in the treated tumors (Figure 7N), which is a well-known feedback mechanism associated with immune activation 34, 35, but also a clear target for rational combination therapy.

### CuZnS@BSA combined with αPD-L1 synergistically enhanced antitumor efficacy

The PD-L1 upregulation observed suggested a clear potential for synergy with immune checkpoint blockade, and since TNBC metastasis is the most recalcitrant aspect of this disease 36, we logically established a lung metastasis model to test CuZnS@BSA+αPD-L1 combination therapy. The results were striking: whereas both monotherapies had some anti-metastatic activity, the combination exerted highly significant metastasis suppression (Figure 8B), confirmed by H&E staining which showed the fewest pulmonary metastatic nodules (Figure 8C) and a 50% reduction in lung weight compared to PBS controls.

From the mechanistic analysis it was clearly and convincingly established that the combination therapy remodels the TIME in lung metastases, with specific and meaningful changes: increased infiltration of mature dendritic cells (Figure 8F-G), reduced M2-like macrophages, increased M1-like macrophages, and hence a substantially elevated M1/M2 ratio (Figure 8H-I). Concurrently, the CD8^+^/CD4^+^ T cell ratio increased (Figure 8J-K). The tumour tissues had increased proportions of Ki67⁺ CD8⁺ and GZMB⁺ CD8⁺ T cells, whereas exhausted Tim⁺ CD8^+^ T cells decreased (Figure 8L-N). Tumor-draining lymph nodes contained more MHC II⁺ dendritic cells (Figure 8O-P), therefore suggesting enhanced antigen presentation, and the spleen showed improved CD8⁺/CD4⁺ T cell ratios with marked relief of T cell exhaustion (Figure 8Q-R). This was elegantly corroborated by Olink proteomic analysis of serum, which detected reduced pro-tumour cytokines and increased anti-tumour cytokines, thus confirming systemic immune activation (Figure 8S).

Since the direct elimination of tumor cells by concurrent apoptosis and cuproptosis is only one aspect, the extensive remodeling of the immune microenvironment actually determines the overall therapeutic outcome, and thus the combined treatment gives the animals a clear survival advantage (Figure 8E). Consequently, the data support very convincingly the clinical applicability of the designed nanoplatform, making it an excellent, practical framework for the treatment of metastatic TNBC.

## Conclusion

From the experimental data collected it is clearly and convincingly established that the therapeutic success of the platform is due to the ordered, coordinated release of copper, zinc, and sulfur ions, which sequentially deplete GSH, induce cuproptosis, and activate STING-mediated immune reprogramming, thereby achieving a therapeutic effect far superior to that of conventional therapies. Thus, the nanoregulator acts as an excellent positive regulator. Because there is a clear feedback loop in which metabolic stress and the ensuing immune response constantly reinforce each other 37, and since the BSA matrix has a simple structure that makes it extremely amenable to large scale manufacturing while retaining outstanding biological compatibility, this mechanism-driven approach represents a very practical strategy for improving clinical outcomes in metastatic TNBC.

The present data unambiguously show that the CuZnS@BSA structure functions as a direct trigger for copper-dependent cell death, with cuproptosis being the dominant mechanism, but there is strong evidence that the nanoregulator induces several overlapping mortality pathways in the target cells. Because targeted inhibitors and specific apoptotic markers have not yet been used to dissect the individual contributions of these parallel pathways to cellular toxicity, future experiments should incorporate specific inhibitors for apoptosis, cuproptosis, and other regulated cell death forms to properly map the regulatory crosstalk. Since the long term immune modulating effects, systemic biosafety, and therapeutic consistency of the platform need to be carefully examined, researchers should extend their *in vivo* studies to a variety of preclinical tumor models.

## Experimental Section

### Materials and reagents

Analytical-grade chemicals were used in all experiments without further purification, and Shanghai Macklin Reagent Co., Ltd. (Shanghai, China) was the source of methylene blue (MB) and bovine serum albumin (BSA). The precursors for the synthesis of the nanoregulators, sodium sulfide nonahydrate (Na_2_S·9H_2_O), zinc acetate dihydrate (Zn(CH_3_COO)_2_·2H_2_O), and anhydrous copper acetate (C_4_H_6_CuO_4_), were also obtained from this supplier. The reagents were purchased from Aladdin Reagent Co., Ltd. (Shanghai, China), while the JC-1 and glutathione (GSH) assay kits came from Solarbio Science and Technology Co., Ltd. (Beijing, China). All the various kits used for cellular and biological evaluations (TUNEL, copper assay, Annexin V-FITC apoptosis, DCFH-DA ROS probe, and Calcein-AM/PI) were obtained from Elabscience Biotechnology Co., Ltd. (Wuhan, China).

Abbkine Scientific Co., Ltd. (Wuhan, China) and Shanghai Maokang Bio Co., Ltd. provided the CCK-8 reagent and the WSP-5 H_2_S probe respectively, while immune cytokines interleukin-4 (IL-4) and granulocyte-macrophage colony-stimulating factor (GM-CSF) were obtained from Novoprotein Scientific, Inc. (Suzhou, China). ELISA kits for the quantitative detection of IFN-γ, IL-4, and TNF-α were obtained from Boster Biological Technology Co., Ltd. (Wuhan, China), while the optical imaging probes (DHE, DiD, Hoechst 33342, and d-luciferin potassium salt) were supplied by Shanghai Shunfeng Biotechnology Co., Ltd. (Shanghai, China). The primary antibodies against LIAS, CRT, DLAT, and FDX1 were all purchased directly from Proteintech (Beijing China). Boster Biological Technology (Wuhan China) provided an additional reagent. n additional panel of primary antibodies directed against CD44, HMGB1, and beta actin was used in this study, so the cell culture media and biological reagents required, namely fetal bovine serum, RPMI 1640, and DMEM, were obtained from Thermo Fisher Scientific (Waltham, Massachusetts, USA). The appropriate secondary antibodies, goat anti rabbit and Alexa Fluor 488 anti mouse conjugates, were purchased from ABclonal Technology (Wuhan, China).

### Cell culture and animal models

The murine 4T1 tumor cells used in this investigation were purchased from Pricella Biotechnology. The female BALB/c mice utilized for *in vivo* experimentation were bought from Wuhan, China from Beijing HFK Bioscience (China, Beijing) aged 6–8 weeks, weighing 18–20g. All animal participants were housed in a designated pathogen-free facility at Jiangnan University additionally, the animal quarters were maintained in strict regulated settings. The environmental parameters were carefully regulated: a 12 h light/dark cycle was maintained ambient temperature was maintained at 22±2 °C, and relative humidity was maintained in the range of 40% – 70%. All mice had ad libitum access to conventional laboratory chow and purified water. Furthermore, all in accordance with established ethical standards, experimental techniques were carried out guidelines, under the direct supervision and formal approval of the Animal Care and Use Committee at Jiangnan University (approval No. JN.20250430b1120930).

### Isolate and culture of BMDCs

Primary bone marrow cells were isolated directly from the hind limbs of female C57BL/6 mice, and BMDCs were induced to differentiate by culturing the isolated cells in RPMI 1640 medium containing 10% heat-inactivated fetal bovine serum, 1% penicillin streptomycin, plus 20 ng/mL granulocyte macrophage colony stimulating factor (GM-CSF) and 10 ng/mL to promote proper lineage development, mL interleukin 4 (IL-4) was added first, and then the cell suspension was seeded into 6 well plates at a density of 2 × 10⁶ cells per mL, followed by incubation in a standard incubator at 37 °C with 5% CO₂ atmosphere. On the third day of incubation, the culture medium was fully replaced to remove non-adherent cell populations. From days 7 to 9, the target BMDCs were carefully harvested based on their characteristic loosely adherent or semi-suspended morphology and were immediately prepared for all subsequent experimental procedures.

### Synthesis and characterization of CuZnS@BSA

The CuZnS@BSA nanoclusters were synthesized by a simple and well-defined biomineralization route: first 40.0 mg of BSA was dissolved in 6.0 mL of deionized water to give a clear aqueous solution, and then 0.5 mL of Zn (CH_3_COO)_2_·2H_2_O (19.25 mg/mL) and 0.5 mL of Cu (CH3COO)_2_·2H_2_O (16.63 mg/mL) were added under continuous stirring. In order to carry out the required sulfidation reaction, 1.0 mL of a Na_2_S·9H_2_O solution (56 mg/mL) was added dropwise, and the mixture was kept at room temperature for 4 h to allow complete reaction. The crude product was then isolated by dialyzing against pure water for 4 h using a membrane with a molecular weight cutoff of 8000–14000. Rotary evaporation was used to concentrate the resulting purified dispersion. The hydrodynamic diameter and surface zeta potential of the product were determined directly and accurately using DLS instrumentation (Malvern Zetasizer Nano ZS United Kingdom). Morphological analysis of the synthesized clusters was performed by TEM (Hitachi HT7700 Japan).

### Assessing hydroxyl radical (•OH) production via MB degradation

To determine the production of hydroxyl radicals (·OH), the bleaching of methylene blue (MB) at 652 nm was measured using a microplate reader. The reaction mixture contained 2 mL of PBS buffer at various pH values, 0.3 mM H_2_O_2_, and 1 mM MB. The Fenton-type reaction was started by adding 100 mg/mL of CuZnS@BSA to the solution, after which the absorption spectra were recorded immediately following a 10 min incubation period. All measurements were carried out in triplicate to ensure data reliability and good reproducibility.

### Testing cell viability

We tested how safe and toxic CuZnS@BSA is to cells. We used a CCK 8 kit for this step. We placed 4T1 cells into 96 well plates. The cells were allowed to attach to the bottom overnight, then the old culture medium was removed and fresh medium containing different amounts of the nanoregulators was added, after which the cells were incubated in the incubator for 6 h. Immediately thereafter, CCK 8 reagent was added to each well, and absorbance was measured at 450 nm using a Bio Rad microplate reader. The cell survival rate was then determined by taking the ratio of the optical density (OD) of the treated groups to that of the untreated control group.

### Calcein-AM and PI cell staining observation

Since the goal was to separate living 4T1 cells from dead cells, the authors appropriately used a Calcein AM and PI dual stain. They seeded exactly 100,000 cells per well in a 12 well plate and allowed the cells to attach for 12 h. Then the planned treatments were applied, and after the treatments were complete, the cells were stained with Calcein AM and PI following the kit manufacturer's protocol precisely. Finally, the cells were examined under a fluorescence microscope to assess toxic effects and morphological changes.

### Assess cell apoptosis

4T1 cells were seeded in 6-well plates at a density of 3 × 10 ⁴ cells/well and cultured for 24 h under standard conditions (37 °C, 5% CO₂). Before performing quantitative assays, the cells were gently dissociated with 0.25% trypsin (EDTA-free, Gibco), washed three times thoroughly with PBS, and then labeled with appropriate fluorescent markers according to conventional protocols. Fluorescence emission profiles were collected by flow cytometry, and the resulting raw data were analyzed using FlowJo software. The method described was used for the assessment of mitochondrial membrane potential.

### Intracellular H_2_S detection

The authors measured intracellular H₂S levels in a very clear and reliable fashion using the fluorescent probe WSP-5: after 6 h of treatment, WSP-5 was added to the culture medium at a final concentration of 15 μM, and the cells were incubated in the dark for 30 min before being washed three times with PBS to remove extracellular background fluorescence. Intracellular H₂S distribution was subsequently examined and quantified by fluorescence microscopy (excitation: 465 nm, emission: 515 nm).

### Assessment of mitochondrial membrane potential

The changes in ΔΨm were measured very carefully with the JC-1 fluorescent probe, so the experiment began with seeding 4T1 cells in 12-well plates at a density of 1 × 10^5^ cells/well and letting them adhere for 24 h under standard culture conditions (37°C, 5%CO₂). After treatment with the nanoregulators cells were stained with JC-1 working solution (30 min, 37 °C) in the dark, followed by fluorescence microscopic analysis of ΔΨm transitions. It is important to note that red fluorescence corresponds to JC-1 aggregates in healthy mitochondria with high MMP, whereas a shift to green fluorescence denotes the production of JC-1 monomers, which is a clear reliable indicator of mitochondrial depolarization (low MMP).

### Measurement of intracellular GSH content

Intracellular GSH fluctuations were monitored by seeding 4T1 cells in 10 cm culture dishes at a well-defined density of 1 × 10⁶ cells per dish, then maintaining the cultures under standard atmospheric conditions (37 °C, 5% CO₂, 24 h) to allow complete surface attachment before dividing them into six separate experimental groups. Internal GSH concentrations were determined by a combination of qualitative imaging and quantitative analysis using specific fluorescent probes together with commercial assay kits. All analytical steps were carried out strictly following the protocols recommended by the manufacturers of the respective reagents.

### Intracellular Cu^2+^ and Zn^2+^ levels in 4T1 cells

The intracellular ion accumulation was studied by seeding 4T1 cells in 12 well plates and dividing the cell population into six well-defined experimental groups, followed by a 6 h therapeutic treatment. Afterward, the cultures were incubated with CSI probes and Zinquin ethyl ester at 37 °C for 30 min, with all steps taken to exclude ambient light. Because the culture surfaces had been thoroughly washed with PBS to eliminate all extracellular background signals, internal ion distributions could be unambiguously imaged by fluorescence microscopy. Therefore, the actual cytosolic Cu²⁺ and Zn²⁺ concentrations were measured using suitable commercial assay kits, with all steps carried out exactly according to the manufacturers' instructions.

### Detection of DLAT oligomerization in 4T1 cells

We seeded 100,000 4T1 cells in confocal dishes to study intracellular protein localization, grew them in a 5 percent CO_2_ incubator at 37 °C for 24 h to allow proper attachment, then applied the experimental treatments. Following this, the cells were fixed with 4 percent paraformaldehyde in PBS and left at room temperature for 30 minutes. Finally, cells were first treated with 0.1% Triton X 100 for 10 minutes, after which 5% BSA was added and the samples were allowed to block non-specific binding for 1 h. Then the anti-DLAT primary antibody was applied at a 1:200 dilution and the dishes were incubated at 4°C overnight. The next morning, Alexa Fluor 488 secondary antibody was added at a 1:500 ratio, and the samples were kept strictly (in the dark, 1 h). Nuclei were stained with Hoechst 33342 (10 min), and high-resolution fluorescence images were finally acquired using a confocal laser scanning microscope.

### mtDNA leakage assay

Mitochondrial DNA translocation was analyzed by culturing 4T1 cells directly on glass coverslips, treating them with the appropriate reagents for 6 h, labeling the intact mitochondrial networks with prewarmed MitoTracker Red, and then maintaining the staining solution at 37 °C for 20–30 min. After thorough rinsing with PBS, the cells were fixed in 4% paraformaldehyde solution for 15 min. The cells were permeabilized with 0.1%Triton X 100 at room temperature for 10–15 minutes, and then cytosolic double stranded DNA was detected by incubating the samples with target-specific fluorescent probes followed by incubation in the dark for 15–30 minutes to allow complete, efficient hybridization. Afterward, the coverslips were mounted on glass slides with antifade mounting medium. The spatial colocalization of mitochondrial remnants with the escaped cytosolic double stranded DNA was therefore unambiguously and systematically analyzed by confocal laser scanning microscopy.

### Transcriptome analysis

The authors systematically characterized the underlying transcriptomic alterations by seeding 4T1 cells, treating them with pure PBS as control or with 20 μg/mL CuZnS@BSA for 24 h, then isolating total cellular RNA using TRIzol reagent according to the manufacturer's protocol. After RNA isolation, the concentration was precisely measured and the RNA integrity was rigorously assessed, with the RNA integrity number determined using a Qubit 2.0 fluorometer. Since all samples gave an optimal RNA integrity number of about 10.0, the fragment size distribution was first reliably determined by the Agilent 4200 TapeStation system. Differentially expressed genes were then analyzed using the DESeq2 package in R, with clear, stringent criteria for statistical significance: an adjusted P value < 0.05 and an absolute fold change ≥ 0.585.

### *In vitro* cuproptosis and cGAS-STING related protein assessment

In order to carry out the cuproptosis and cGAS-STING protein analyses, we cultured 4T1 cells in six-well plates and seeded them at 1 × 10^5^ cells per well, allowing the cells to attach firmly for 12 h before subjecting them to 24 h of nanoplatform treatment at 37°C. After treatment, the cell monolayer was rinsed with cold PBS, the cells were harvested by gentle collection, and total proteins were then extracted using RIPA with 1% protease inhibitors, kept them chilled on ice for 30-60 min, and then cleared the cellular debris by centrifugation at 12000 × g, 15 min, 4 °C. The total protein concentration was accurately determined using an Abbkine BCA kit. Prior to loading, the protein samples were mixed with 4 × SDS loading dye in a ratio of 3:1 the proteins were first treated with 2% SDS and boiled for 10 min at 95°C, then equivalent protein amounts were loaded onto 8% SDS-PAGE gels. Electrophoresis was carried out at 120 V, 100 min, followed by a wet transfer step in which the resolved bands were transferred onto Millipore 0.22 μm PVDF, 300 mA, 90 min. To minimize non-specific background signals, the membranes were soaked in a 5% skim milk TBST solution for 2 h (room temperature). After blocking, the membranes were incubated overnight at 4 °C with the relevant primary antibodies. The next day, HRP-conjugated secondary antibodies (diluted 1:5000) were added 1 h. Chemiluminescence was induced with ECL reagents, and all digital blot images were collected using the Shanghai Tanon system.

### Bio-TEM observation

We systematically examined the effect of the nanoregulator on mitochondrial morphology by first incubating 4T1 cells with pure PBS to serve as a control or with 20 μg/mL CuZnS@BSA in pH 6.0 buffer for exactly 12 h, then fixing the cells with 2.5% glutaraldehyde solution at room temperature for 10 min. After preparing nanoscale sections by standard ultramicrotomy techniques, the ultrastructure and precise morphological changes of the mitochondria were directly and clearly analyzed using biological transmission electron microscopy (bio TEM).

### *In vitro* studies of ICD

For the *in vitro* ICD evaluation, we initially cultured 4T1 cells on coverslips inside 12-well plates and gave them 12 hours at 37 °C to attach. We then applied the various test formulations for a 24-hour period. Afterward, we recovered the culture media and centrifuged it for 10 minutes at 1000 × g (4 °C) to obtain a clear supernatant. We fixed the remaining cells with 4 percent paraformaldehyde at room temperature for 15 minutes. Our staining preparation depended on the target: we blocked directly with 5 percent BSA for CRT, but we added a 0.2 percent Triton X-100 permeabilization step before blocking for HMGB1. We probed the samples using anti-CRT or anti-HMGB1 primary antibodies at 4 °C overnight. Following this, we introduced FITC-conjugated secondary antibodies and counterstained the cell nuclei using Hoechst. We took all images with a Zeiss Axio Vert A1 fluorescence microscope and performed the intensity calculations through ImageJ.

### Analysis of DC activation* in vitro*

The effect of nanoregulators on BMDC maturation was conveniently and rigorously assessed using a Transwell system: 4T1 cells were first pre-treated with nanoclusters for 6 h, then co-incubated with BMDCs seeded in the bottom compartment of the Transwell insert. Following co-culture, BMDCs were harvested, fixed if necessary, and stained with a fluorescent antibody cocktail consisting of PE-conjugated anti-mouse CD11c, FITC-conjugated anti-mouse CD80, and APC-conjugated anti-mouse CD86 (Elabscience, China). After 30 min of incubation to allow optimal antibody binding, marker expression levels were analyzed by BD Celesta flow cytometry.

### *In vivo* biosafety assessment

Biosafety was rigorously evaluated using healthy female BALB/c mice (18–22 g, 6–8 weeks; n = 6–8/group), with major organs harvested after sacrifice and processed for histopathological analysis: tissues were fixed in 4% paraformaldehyde, paraffin-embedded, sectioned at 5 μm, and then stained with H&E. Systemic toxicity was assessed by serum biochemical analysis, which involved centrifuging the blood samples at 3,000 × g for 15 min, followed by automated biochemical analysis of the serum to measure standard markers of hepatic and renal function, namely ALT, AST, ALP, urea, and CREA.

### Hemolysis assay

To evaluate blood compatibility, a standard hemolysis experiment was carried out using rat red blood cells (RBCs), with erythrocytes first suspended in PBS to prepare a 4% (v/v) stock solution, which was stored at 4°C. PBS and 1% (v/v) Triton X-100 were used as negative and positive controls, respectively. For the assay, 500 μL of the 4% RBC suspension was mixed with 500 μL of nanoregulator solutions of varying concentrations in 1.5 mL tubes, then incubated at 37°C for 4 h with gentle periodic shaking. After centrifugation at 3,000 rpm for 5 min, the supernatants were collected. Hemoglobin release was first assessed qualitatively by photography and then quantified by measuring absorbance at 540 nm on a microplate reader. The hemolysis rate was therefore calculated straightforwardly from these data.

Hemolysis (%) = 
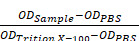
×100

### Tumor model establishment

The subcutaneous 4T1 tumor model was established in female BALB/c mice by first sterilizing the lateral thigh skin with 75% ethanol, inserting a 1 mL syringe needle into the subcutaneous tissue at a 45° angle to a depth of about 1 cm, aspirating to verify proper placement, and then injecting a suspension of 1.5 × 10^6^ luciferase-expressing 4T1 cells slowly. After injection, the site was briefly compressed to prevent leakage. For the breast cancer lung metastasis model, mice were injected via the tail vein with 0.5 × 10^6^ luciferase-expressing 4T1 cells. Tumor progression was then monitored non-invasively using an IVIS Spectrum system (PerkinElmer) by bioluminescence imaging (BLI) after intraperitoneal administration of D-luciferin (150 mg/kg). The number of metastatic nodules was manually counted by two independent pathologists on H&E-stained whole-lung sections, and the mean value was taken to supplement the deficiencies of gross observation.

### Biodistribution and tumor targeting analysis

To evaluate systemic distribution and tumor targeting, CuZnS@BSA-NHS-Cy7 was administered intravenously to tumor-bearing mice, and longitudinal biodistribution was analyzed by real-time fluorescence imaging (IVIS Spectrum, PerkinElmer; Ex/Em:710/760 nm) at 6, 12, 24, and 48 h after injection. At 24 h, the mice were sacrificed for *ex vivo* imaging, and major organs (heart, liver, spleen, lungs, and kidneys) as well as tumor tissue were harvested, after which fluorescence signal intensity was quantified using the IVIS Spectrum system.

### Serum cytokine quantification

In order to evaluate therapeutic efficacy, female BALB/c mice (6 weeks old) were used to establish the 4T1 subcutaneous model, and when the tumor volume had reached the target range of 75–100 mm³, the tumor-bearing animals were randomly divided into five groups (n = 10 per group for survival analysis): (1) PBS (control), (2) αPD-L1 (i.p.), (3) CuZnS@BSA (i.v.), and (4) combination therapy (αPD-L1 i.p. + CuZnS@BSA i.v.). After completion of the 14-day treatment regimen, mice were sacrificed for histological and immunological analysis, and primary tumors as well as pulmonary metastatic nodules were excised for immunohistochemical validation (H&E, Ki-67, and TUNEL). At the same time, major viscera, lymph nodes, and serum were collected for biosafety evaluation. The immune landscape of the spleen, tumor-draining lymph nodes, and tumor tissues was systematically analyzed by flow cytometry and immunofluorescence, and overall survival was followed for 60 days post-treatment.

### ELISA for cytokine profile analysis

The concentrations of inflammatory cytokines (IFN-γ, TNF-α, and IL-6) in serum were determined in order to assess the systemic immune response, and this was done by using commercial ELISA kits following the manufacturer's protocols. Blood was collected from the orbital sinus of the mice, and after incubation at room temperature for 60 minutes to allow clotting, the samples were processed. Serum was separated by centrifugation at 3000 rpm and 4 °C for 20 min, and the resulting serum samples were assayed in duplicate. The absorbance of the reaction products was measured at 450 nm with a microplate reader. Target cytokine concentrations were then calculated by interpolating measured optical density values against a standard curve derived from recombinant protein standards.

### Olink for cytokine profile analysis

Since high-throughput quantification of serum cytokines was the goal, the Olink Target 48 Cytokine Panel (Olink Proteomics AB, Uppsala, Sweden) was used exactly as specified by the manufacturer. More importantly, the Proximity Extension Assay (PEA) platform employed here follows the well-described methodology of Assarsson *et al*.(2014) and therefore enables reliable simultaneous measurement of 92 different analytes from as little as 1 µL of sample.Protein-specific antibody probes conjugated with complementary DNA oligonucleotides recognize the target analytes in this workflow, and hybridization of the oligonucleotides upon close proximity initiates a DNA polymerase-mediated extension reaction, thus generating sequence-specific PCR amplicons. The resulting synthetic DNA products were then quantified with the Signature Q100 microfluidic real-time PCR system. The R instrument (Hangzhou Lianchuan Biotechnology Co., Ltd., Hangzhou, China) was used in the experiment, and to obtain reliable quantitative results, all datasets were first properly calibrated against internal and inter-plate controls to eliminate experimental variation. Since the final abundances were expressed as Normalized Protein expression (NPX) values, which are arbitrary units on a log2 scale that have a direct linear correlation with protein concentration, it is appropriate to refer to the detailed analytical performance data for assay sensitivity and precision on the Olink portal (www. olink. com).

### Statistical analysis

The data were analyzed with GraphPad Prism (version 10.0) and are presented as mean ± standard deviation (SD) based on at least three independent replicates. Statistical significance between two groups was assessed by unpaired two-tailed Student's t-test, whereas comparisons among multiple groups were done using one-way ANOVA followed by Tukey’s post hoc test. The level of significance is accordingly indicated as: ^*^P < 0.05,^ **^P < 0.01, ^***^P < 0.001, ^****^P < 0.0001, and n.s. (not significant).

## Supplementary Material

Supplementary figures and tables.

## Figures and Tables

**Scheme 1:**
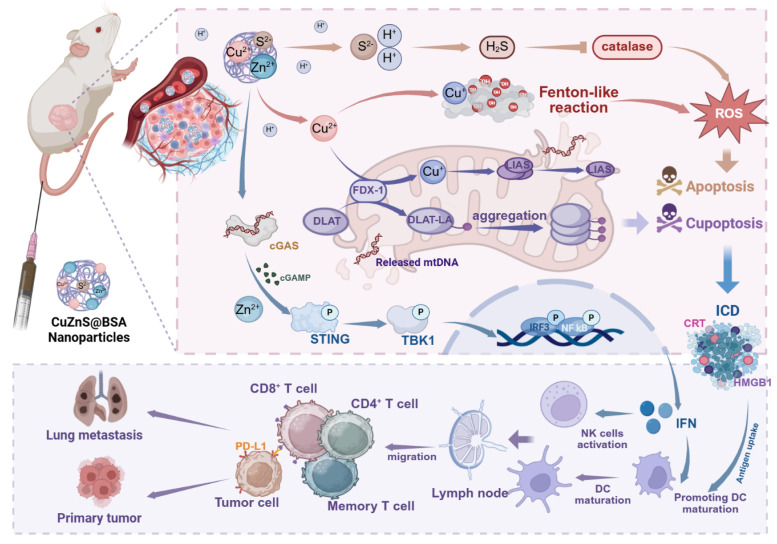
The therapeutic mechanism of the CuZnS@BSA nanoplatform.
