# Supplementary material for: Metabolic-Immune Reprogramming via CuZnS@BSA Nanoregulators to Overcome Resistance in Triple-Negative Breast Cancer
Source: Theranostics. 2026 Jul 22;16(14):8263–83. doi: 10.7150/thno.134131 (PMC13440635; doi:10.7150/thno.134131)
Supplement: Supplementary file 1 [file thnov16p8263s1.pdf]

# Metabolic-Immune Reprogramming via CuZnS@BSA Nanoregulators to Overcome Resistance in Metastatic Triple-Negative Breast Cancer

**Authors**

*Jingyi Yang[#]; Qi Li[#]; Pi Zhao[#]; Zixin Luo; Zhaokai Wang; Rui Yang; Min Zhou; Jie Zhou; Daozhen Chen[*]; Tao Wan[*]; Yu Chen[*]*

**Affiliations**

J. Yang, Q. Li, R. Yang, M. Zhou, J. Zhou, D. Chen, Y. Chen
Affiliated Women's Hospital of Jiangnan University, Wuxi School of Medicine, Jiangnan University, Wuxi 214002, China
Email: chendaozhen@163.com; cy-78@hotmail.com;

D. Chen
Affiliated Women's Hospital of Jiangnan University, Wuxi School of Medicine, Jiangnan University, Wuxi 214002, China
Wuxi Higher Health Vocational Technology School,
Wuxi, Jiangsu 214028, China

P. Zhao, Z. Luo, Z. Wang, T. Wan
Wuxi School of Medicine, Jiangnan University, Wuxi, Jiangsu 214122, China
Email: 8202304002@jiangnan.edu.cn

*Corresponding author.
[#] These authors contributed equally to this work.

**Funding**

This study was supported by the National Natural Science Foundation of China (82473350; 82404536); Major scientific research project of Wuxi Municipal Health Commission (Z202408); Jiangsu Provincial Natural Science Foundation (BK20241626); Wuxi Municipal Science and Technology Development Fund Project (K20253012); Youth Scientific Research Project of Wuxi Municipal Health Commission (Q202530).

**Keywords**

Triple-negative breast cancer; Cuproptosis; Immunogenic cell death; Stimulator interferon genes (STING); Cancer immunotherapy

## Supporting Information

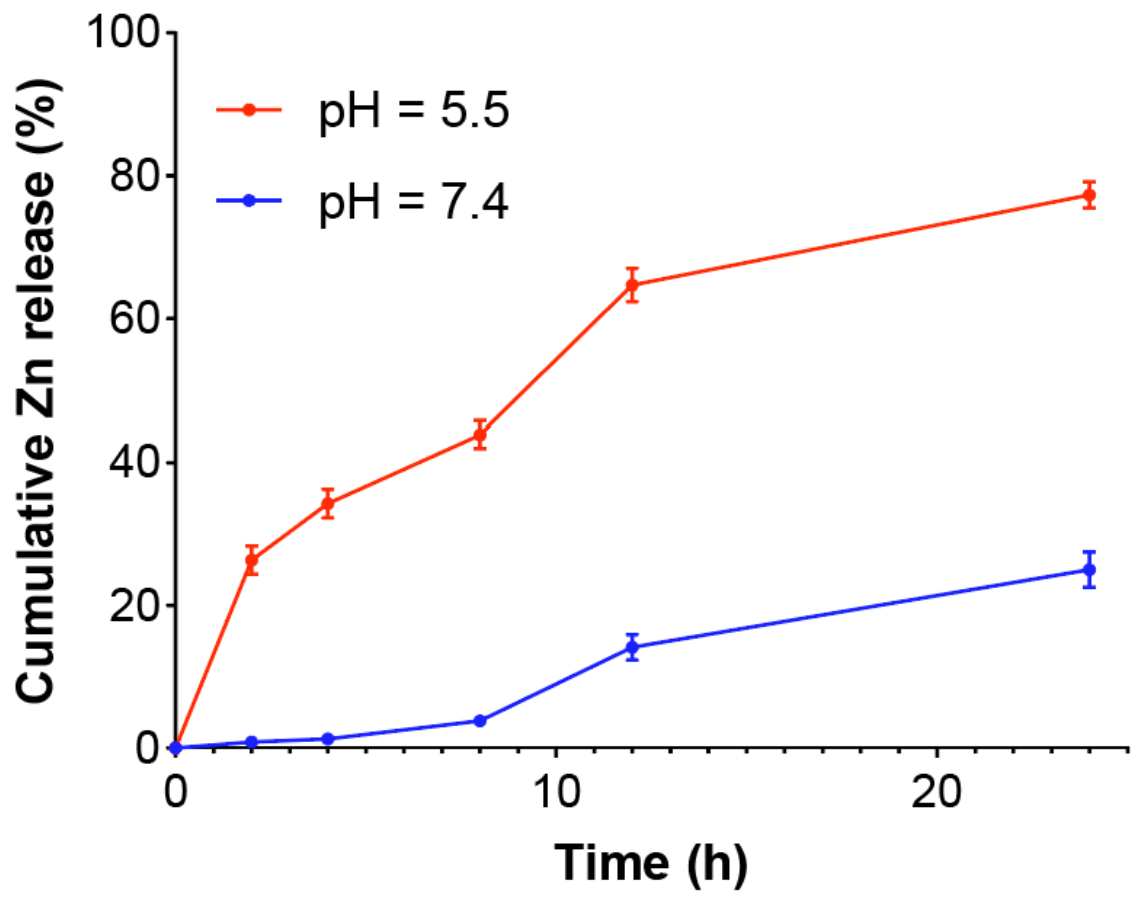


**Figure S1. Cumulative release of Zn from CuZnS@BSA at different pH values (*n* = 3 independent samples, mean ± SD).**

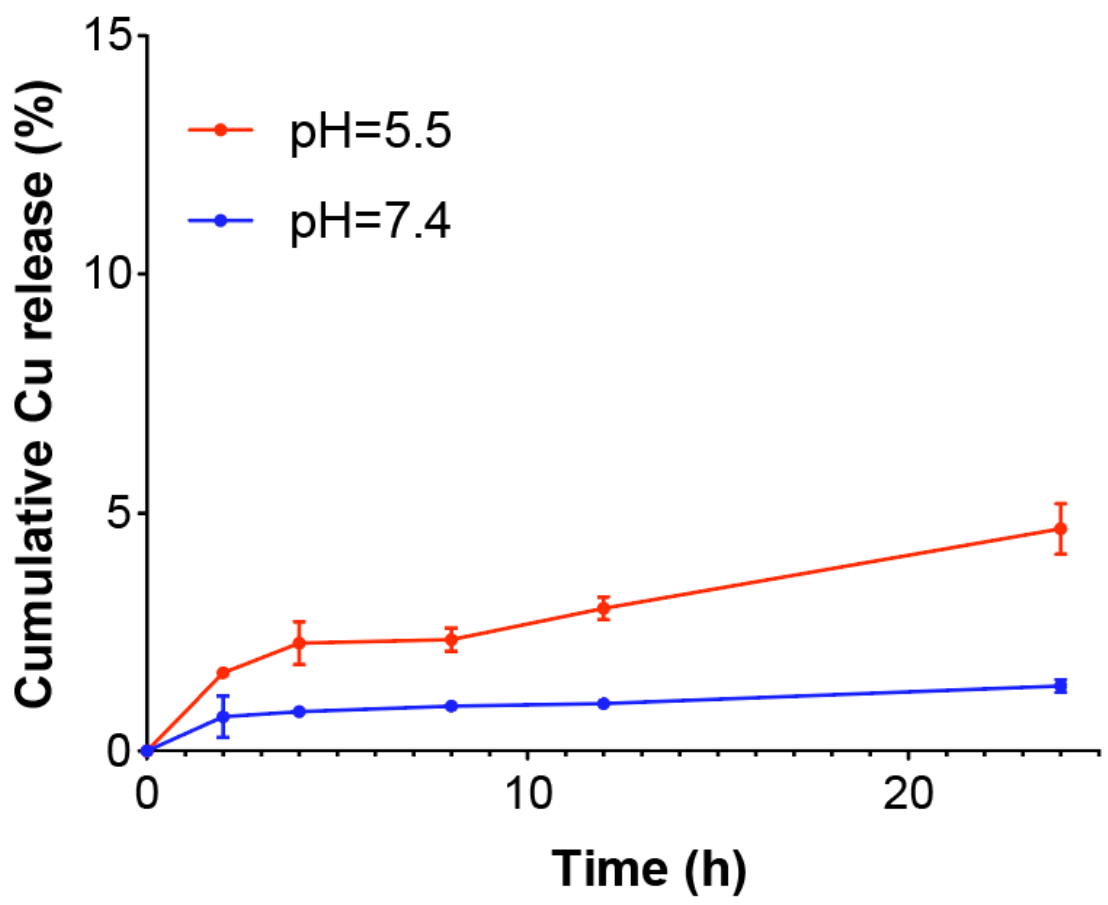


**Figure S2. Cumulative release of Cu from CuZnS@BSA at different pH values (*n* = 3 independent samples, mean ± SD).**

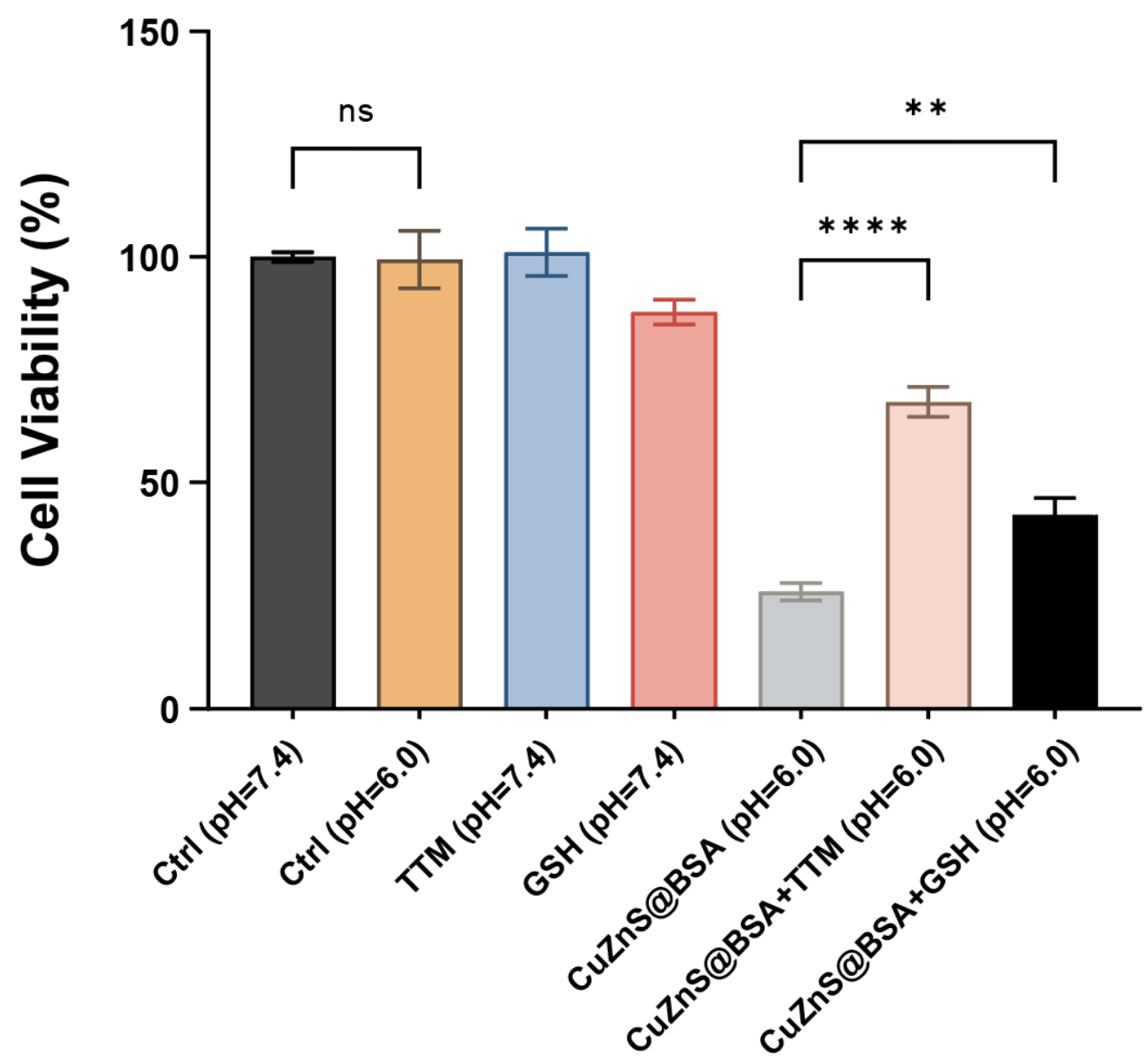


**Figure S3. Relative viability of 4T1 cells treated with CuZnS@BSA in the presence or absence of GSH or TTM (*n* = 3 independent samples, mean ± SD, Statistical significance was determined using one-way ANOVA followed by Tukey's post hoc test. ** *P* < 0.01, **** *P* < 0.0001, ns = not significant.).**

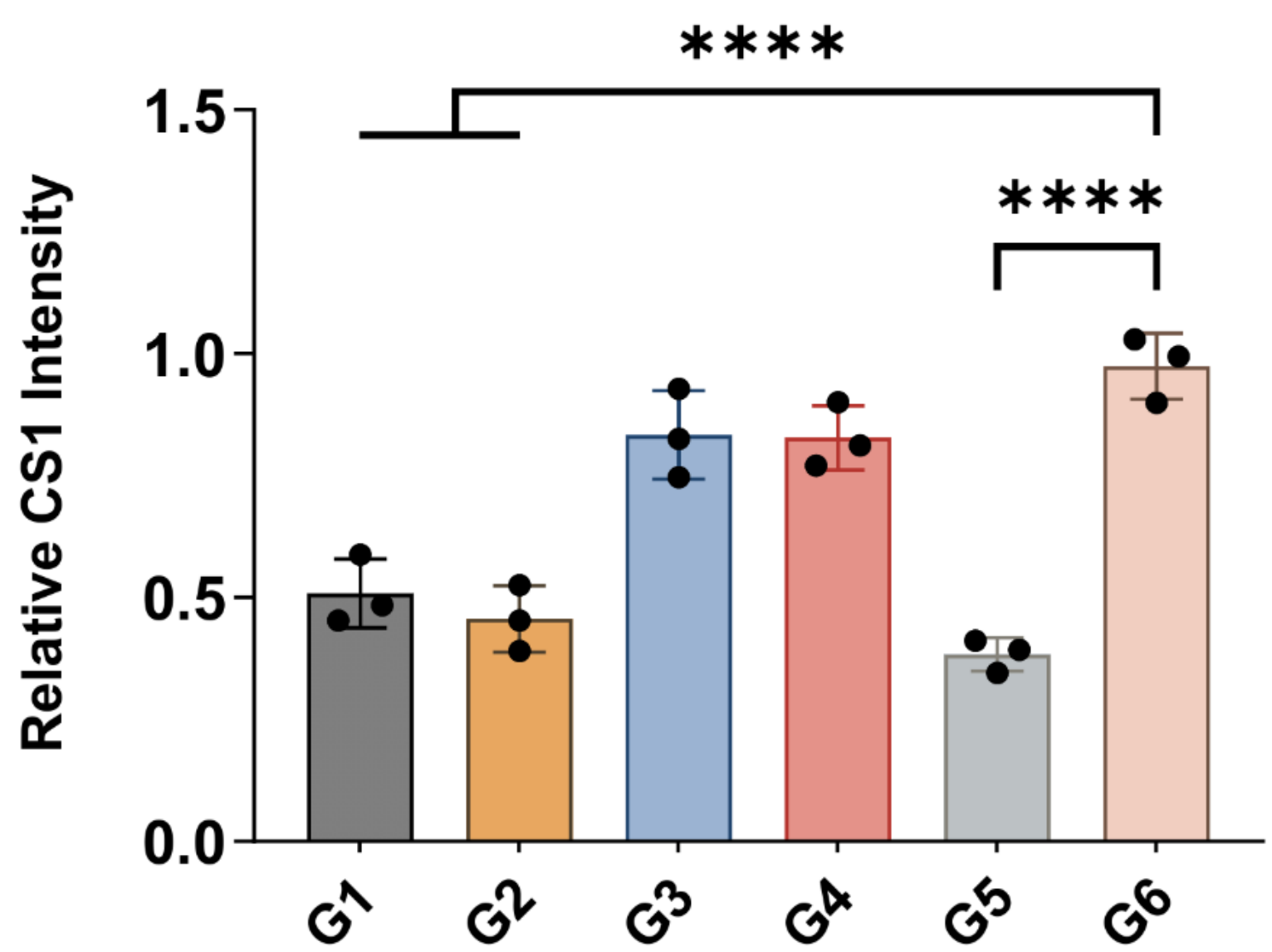


**Figure S4. Relative intracellular copper levels in 4T1 cells following various treatments.**

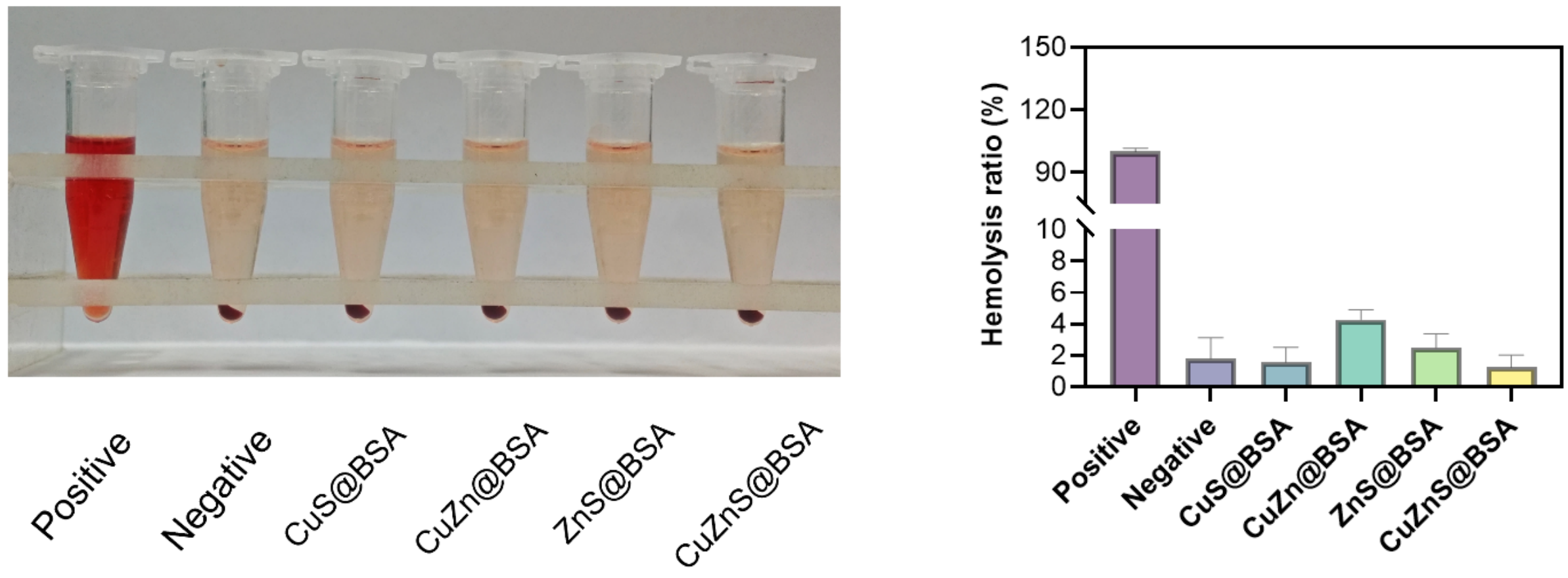


**Fig. S5. Hemolytic test of different groups (*n* = 3 independent samples, mean ± SD).**

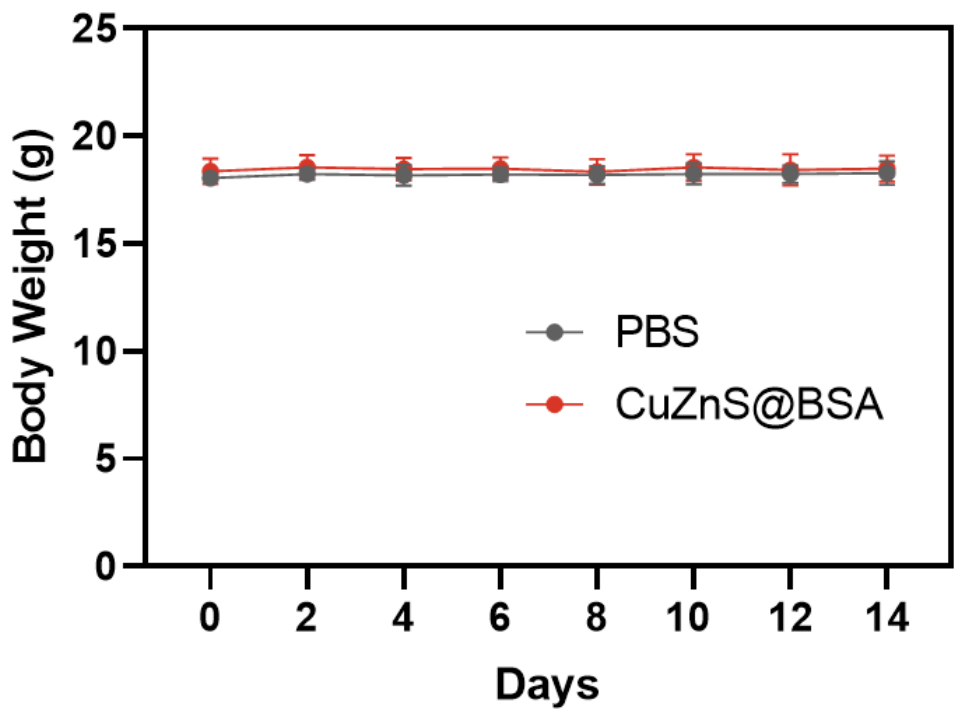


**Fig. S6. Body weight changes in healthy mice after intravenous injection CuZnS@BSA (*n* = 4 independent samples, mean ± SD).**

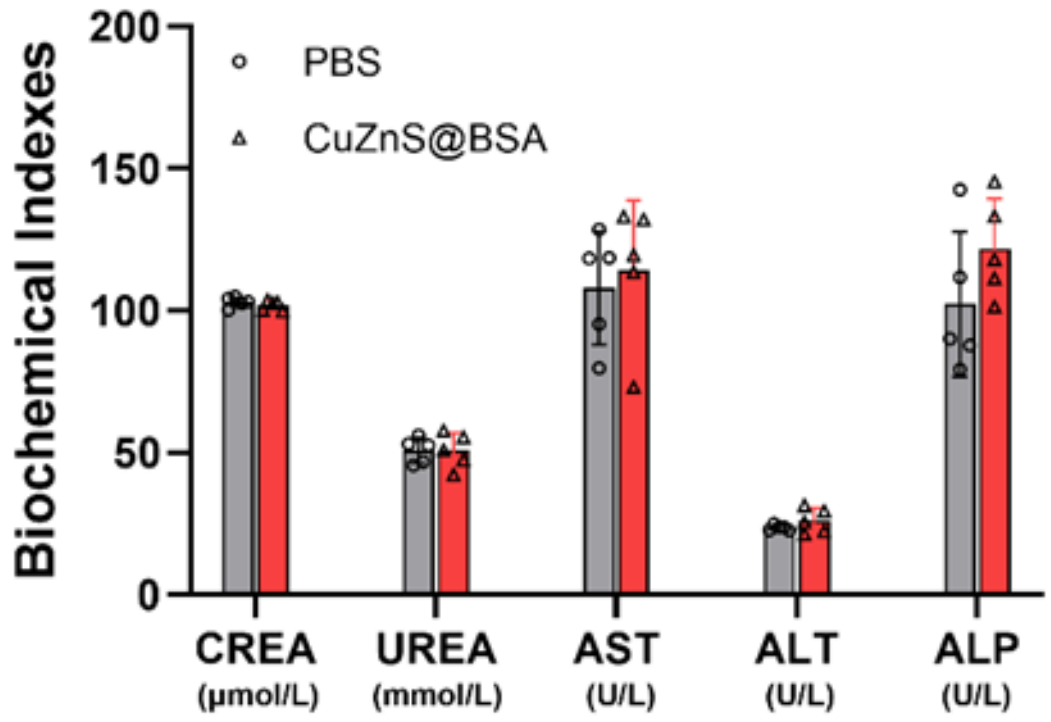


**Fig. S7.Kidney function indicators (CREA, UREA) and liver function indicators (AST, ALT, ALP) on days14 (*n* = 5 independent samples, mean ± SD).**

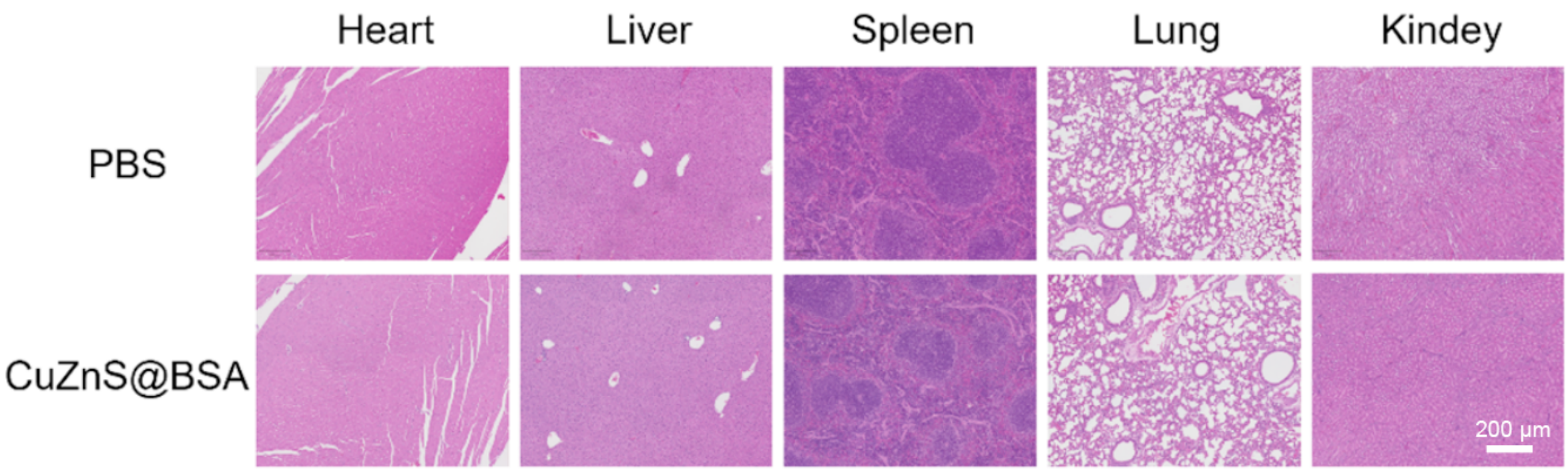


**Fig. S8. H&E-stained images of major organs (*n* = 3 independent samples).**

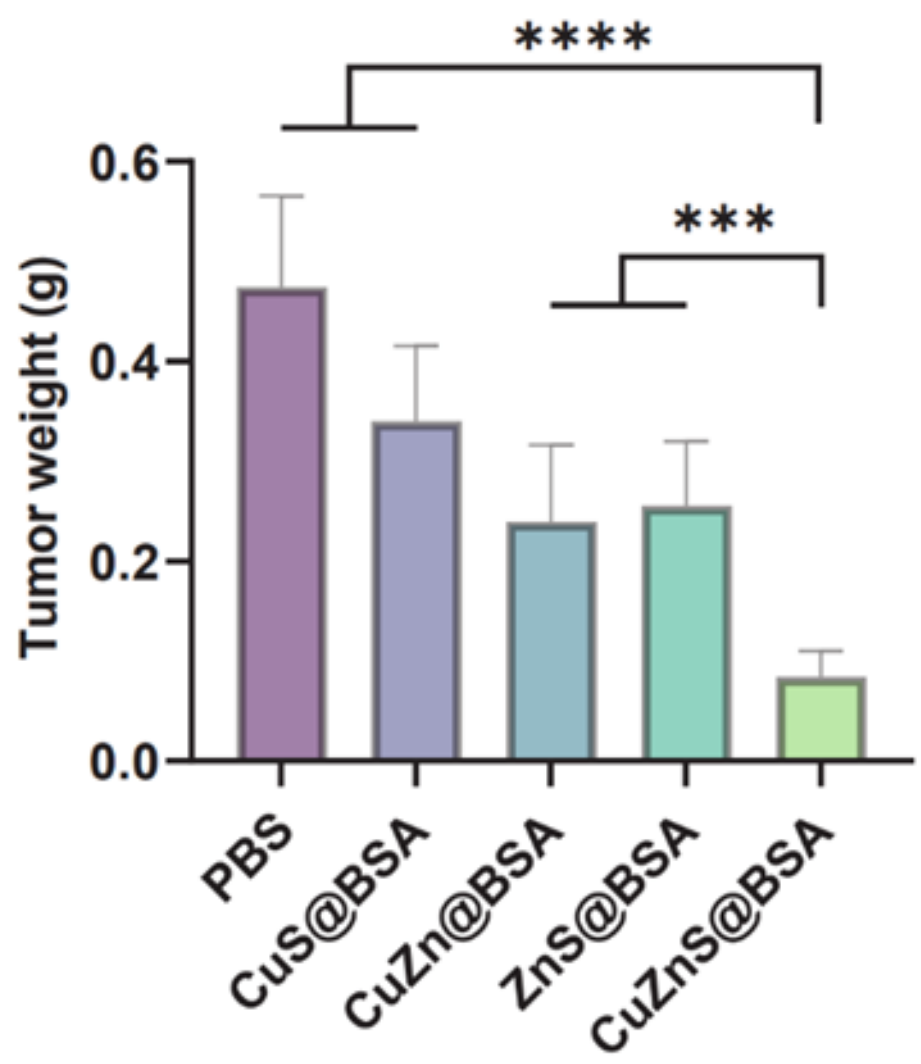


**Fig. S9. Tumor weight at day 21 in five groups (*n* = 6 independent samples, mean ± SD).**

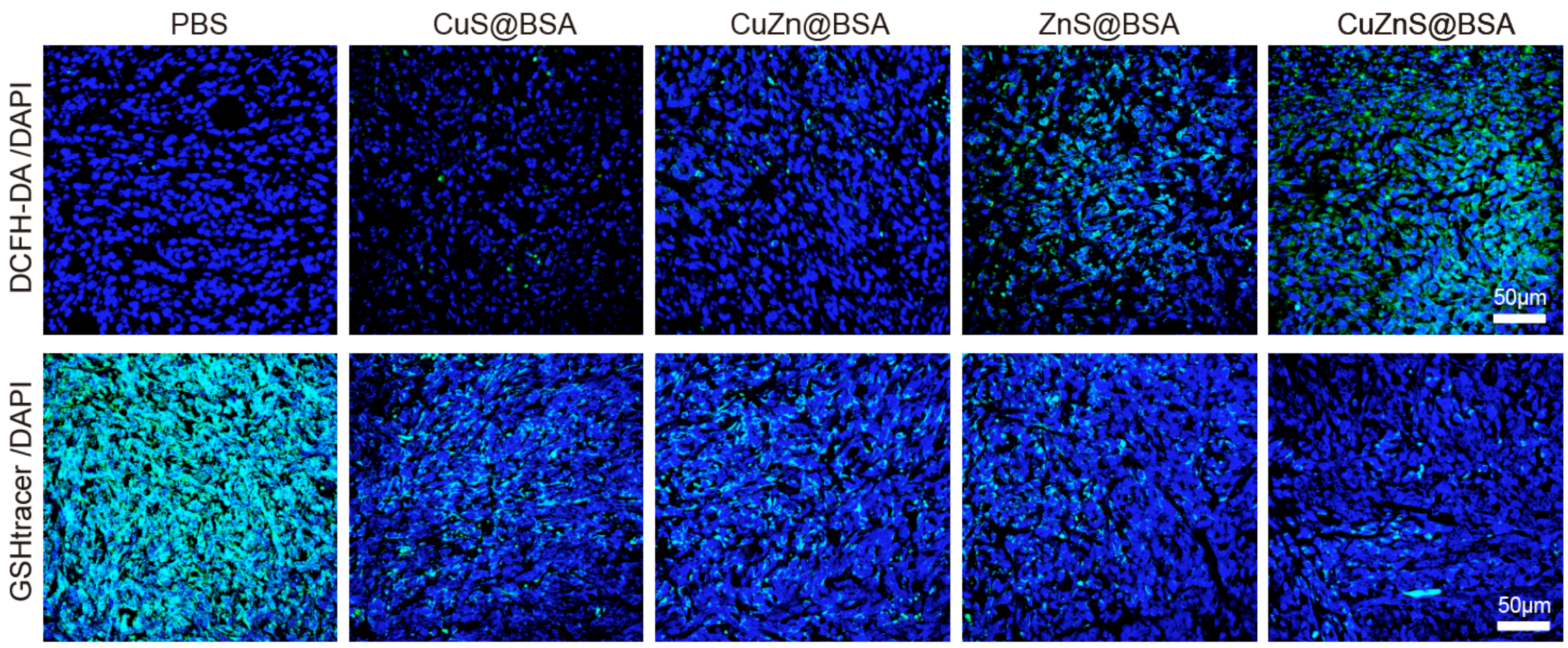


**Fig. S10. ROS and GSH staining of tumor tissues with different treatments (*n* = 3 independent samples).**

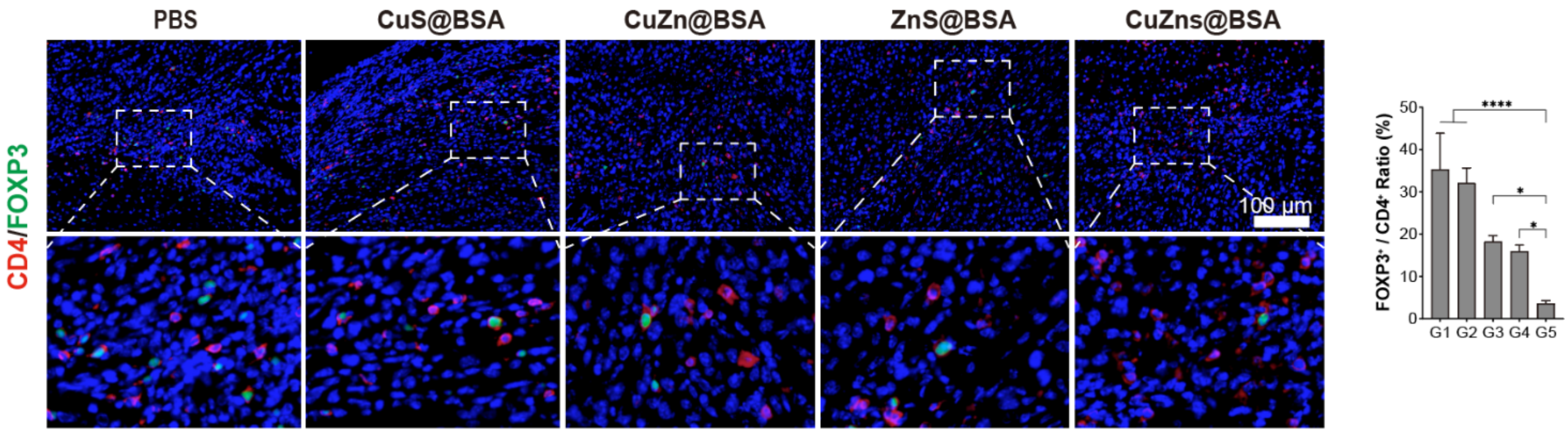


**Fig. S11. Depletion of immunosuppressive regulatory T cells (Tregs) of tumor tissues with different treatments (*n* = 3 independent samples). Statistical significance was determined using one-way ANOVA followed by Tukey's post hoc test. * $P < 0.05$, **** $P < 0.0001$.**

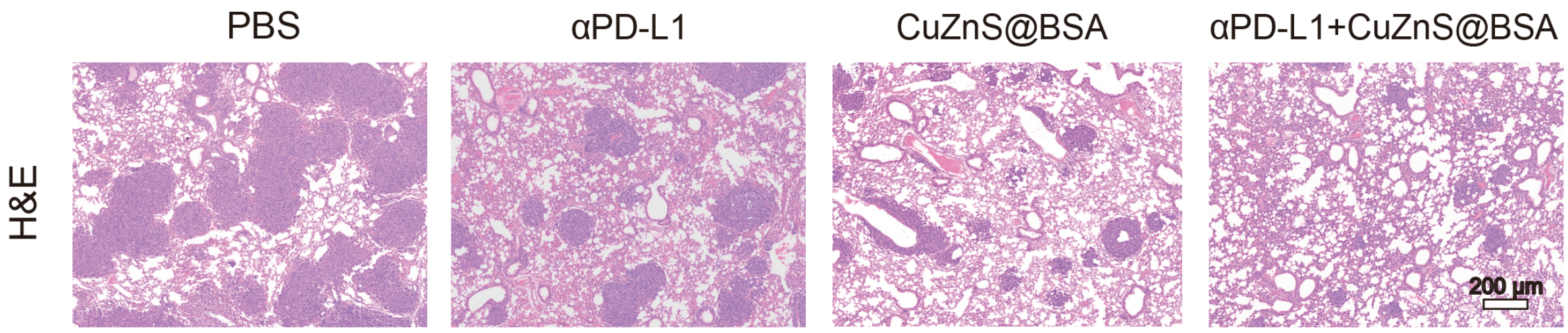


**Fig. S12. H&E of lung tissue with different treatments (*n* = 3 independent samples).**

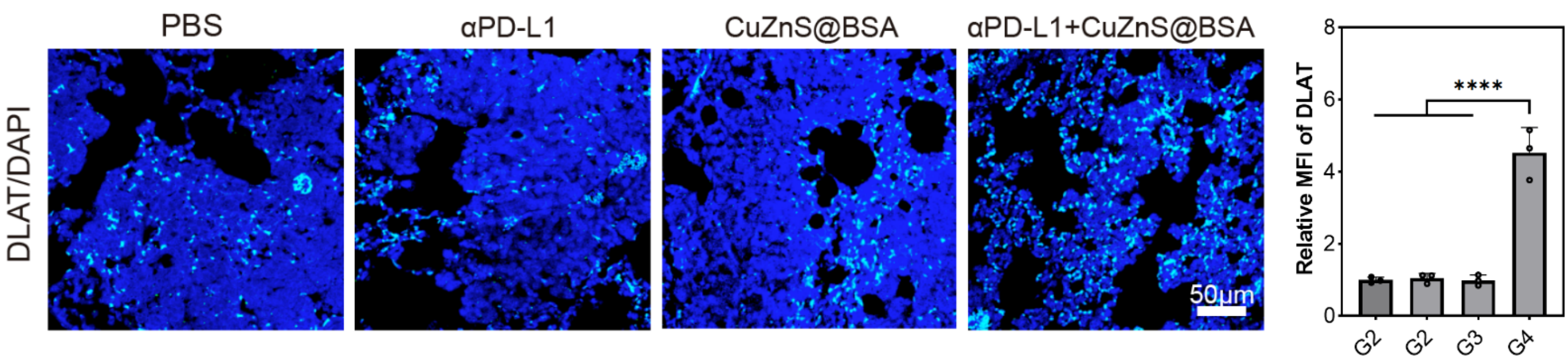

**Fig. S13. DLAT immunofluorescence staining of lung tissue after different treatments (n=3 independent samples).**

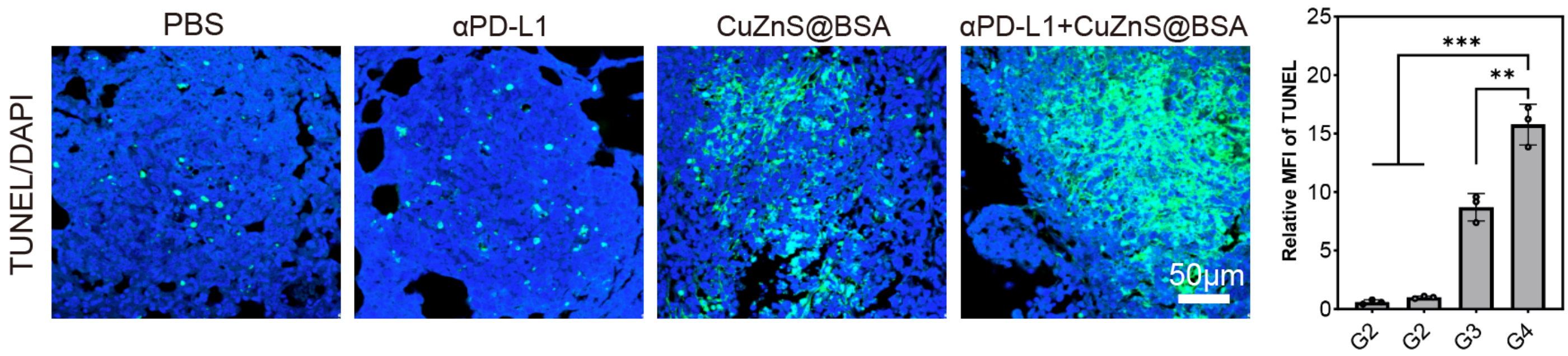


**Fig. S14. TUNEL fluorescence staining of lung tissue after different treatments (n=3 independent samples).**

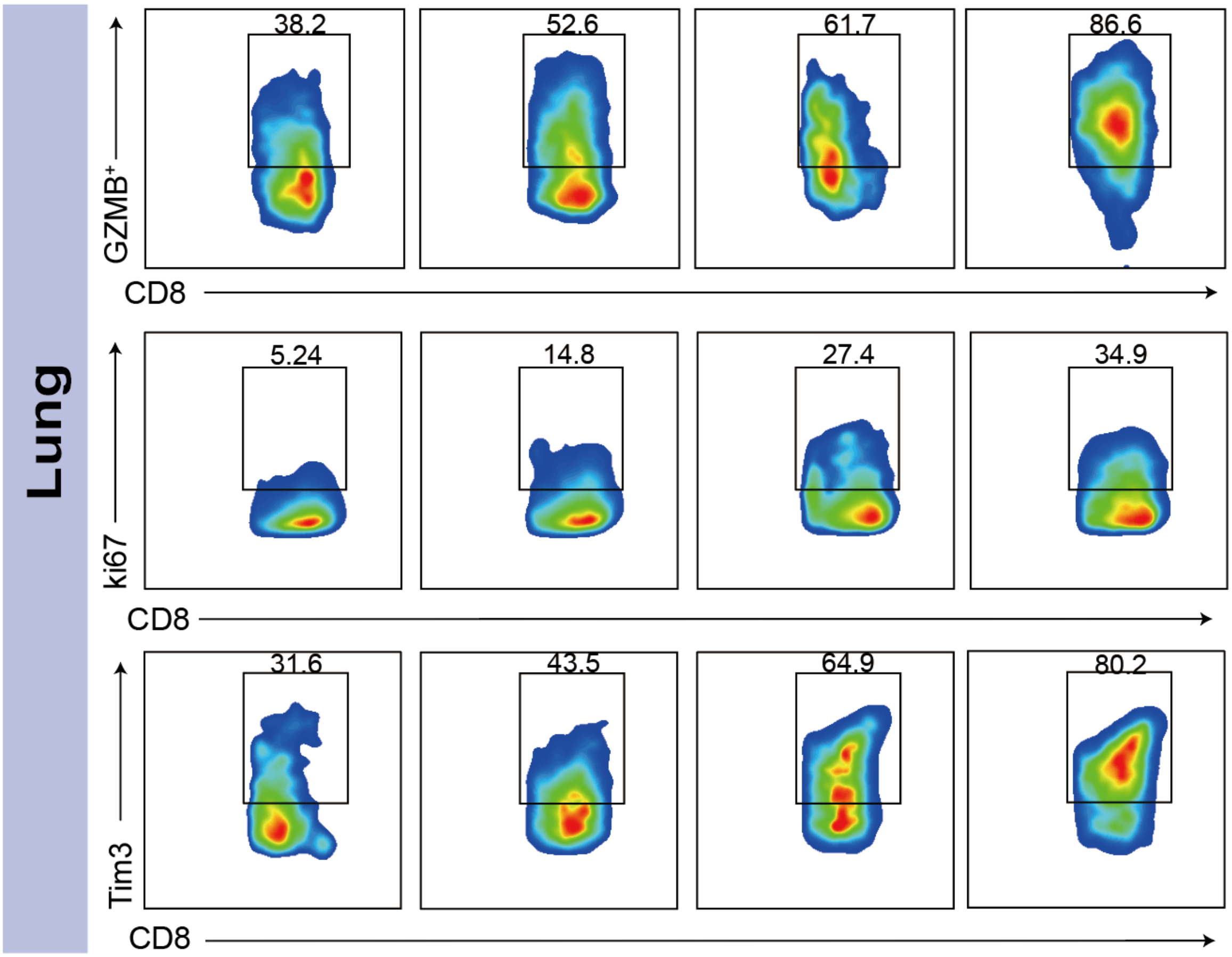


**Fig. S15. Quantitative assessment of GzmB+, Ki67+, and Tim3+ expression levels in CD8+ T cells extracted from lung tissue using flow cytometry.**
